# *In silico* Evolutionary Divergence Analysis Suggests the Potentiality of Capsid Protein VP2 in Serotype-Independent Foot-and-Mouth Disease Virus Detection

**DOI:** 10.3389/fvets.2020.00592

**Published:** 2020-09-25

**Authors:** Israt Dilruba Mishu, Salma Akter, A. S. M. Rubayet Ul Alam, M. Anwar Hossain, Munawar Sultana

**Affiliations:** ^1^Department of Microbiology, University of Dhaka, Dhaka, Bangladesh; ^2^Department of Microbiology, Jahangirnagar University, Dhaka, Bangladesh; ^3^Department of Microbiology, Jashore University of Science and Technology, Jashore, Bangladesh

**Keywords:** FMDV, VP2 vs. VP1, evolutionary divergence, serotype-independent, conserved sites, consensus VP2 protein, B cell epitope

## Abstract

Foot-and-mouth disease (FMD) is an economically devastating disease of the livestock worldwide and caused by the FMD virus (FMDV), which has seven immunologically distinct serotypes (O, A, Asia1, C, and SAT1–SAT3). Studies suggest that VP2 is relatively conserved among three surface-exposed capsid proteins (VP1–VP3) of FMDV, but the level of conservation has not yet been reported. Here we analyzed the comparative evolutionary divergence of VP2 and VP1 to determine the level of conservation in VP2 at different hierarchical levels of three FMDV serotypes (O, A, and Asia1) currently circulating in Asia through an in-depth computational analysis of 14 compiled datasets and designed a consensus VP2 protein that can be used for the development of a serotype-independent FMDV detection tool. The phylogenetic analysis clearly represented a significant level of conservation in VP2 over VP1 at each subgroup level. The protein variability analysis and mutational study showed the presence of 67.4% invariant amino acids in VP2, with the N-terminal end being highly conserved. Nine inter-serotypically conserved fragments located on VP2 have been identified, among which four sites showed promising antigenicity value and surface exposure. The designed 130 amino acid long consensus VP2 protein possessed six surface-exposed B cell epitopes, which suggests the possible potentiality of the protein for the development of a serotype-independent FMDV detection tool in Asia. Conclusively, this is the first study to report the comparative evolutionary divergence between VP2 and VP1, along with proposing the possible potentiality of a designed protein candidate in serotype-independent FMDV detection.

## Introduction

Foot-and-mouth disease (FMD) is a highly contagious disease of cloven-hoofed animals, causing a large-scale economic loss for livestock industries worldwide due to the rapid loss of productivity (1–3). The onset of FMD can cause extensive morbidity and mortality, resulting in a disastrous reduction in the yield of animal products. The etiologic agent, FMD virus (FMDV), is a member of the *Picornaviridae* family, possessing a single-stranded positive-sense RNA genome (4–6), which encodes four structural proteins (VP1–VP4) and other non-structural proteins (Lpro, 2A, 2B, 2C, 3A, 3B, 3Cpro, and 3Dpol).

Extensive mutational variations result in the differentiation of the virus into seven immunologically distinct serotypes; O, A, C, Asia1, and Southern African territories (SAT) 1–3 (7). Within serotypes, there are multiple topotypes that are usually related to the geographical region of disease occurrence or subtype and being identified based on a threshold of 15% nucleotide sequence divergence in the VP1 coding region. However, the high genetic diversity of the virus results in the emergence of many distinct lineages within a topotype, which show at least 7.5% VP1 nucleotide divergence (8, 9). Further diversification divides the individual lineages into multiple sub-lineages, although there is no established threshold for VP1 divergence among these sub-lineages.

Due to the relative ease of sequencing and the reliability of virus classification, VP1-based molecular epidemiology has been accepted as the traditional way of evolutionary divergence analysis. Studies suggest that VP1 is the most diversified capsid protein of FMDV at both nucleotide and amino acid (aa) levels (10–12), but there is lack of information regarding the extent of VP1 divergence in comparison with other capsid proteins. Comparative evolutionary divergence analysis can provide us with information regarding the possible chance of mutation and evolutionary stability of two different proteins. This information can be used for selecting the most conserved one for the future development of a more reliable diagnostic tool that will be capable of detecting FMDV regardless of its serotypes. Among the three surface-exposed capsid proteins of FMDV (VP1–3), VP2 is reported to be relatively conserved (10) and to also possess potential immunogenic sites capable of eliciting the development of anti-VP2 antibody upon infection (13). That is why VP2-based evolutionary divergence of FMDV has been investigated in the current study and has been compared with VP1-based divergence at multiple taxonomic hierarchies (serotypes, topotypes, lineages, and sub-lineages).

Overall, the current study aims at analyzing the comparative evolutionary divergence of VP2 and VP1, along with identification of the surface-exposed conserved antigenic sites in VP2 and designing a consensus VP2 protein as a potential candidate to develop a rapid and cost-effective tool for FMD diagnosis in the Asian region, which would be entirely serotype independent. Herein we have analyzed the molecular diversity of VP2 at both genomic and proteomic levels, considering multiple taxonomies (*i*. *e*., serotypes, topotypes, lineages, and sub-lineages), and identified the surface-exposed conserved antigenic sites in VP2. Based on these findings, we have designed a consensus VP2 protein sequence of 130 amino acid long and mapped possible B cell epitopes in this protein. To our present knowledge, this study is the first one to report on VP2-based evolutionary divergence cutoff values of FMDV at each subgroup and to design a consensus VP2 protein carrying surface-exposed B cell epitopes to be used in serotype-independent FMD diagnosis.

## Materials and Methods

### Sequencing of VP2 Coding Region

Viral RNA extraction and cDNA preparation of 20 local FMDV isolates were performed from cell culture supernatants of selected isolates using Maxwell® 16 Viral Total Nucleic Acid Purification Kit (Promega, USA) and GoScript™ Reverse Transcription System (Promega, Madison, WI, USA) according to the manufacturer's protocol. The reverse-transcribed cDNA samples were used for amplification of the VP2 coding regions by the designed primers VP2-F (GACAAGAAAACCGASGAGACCAC) and VP2-R (TCTTTGGAAKGGAACTCACSCG). After purification of the amplified PCR products, sequencing of the desired VP2 coding region was performed using Sanger sequencing method, and the obtained sequences were analyzed with SeqMan (14). The sequences were submitted to NCBI, and the accession IDs are listed in Supplementary Table 1g.

### Dataset Generation and Compilation

Three major FMDV serotypes circulating in Asian regions (O, A, and Asia1) were focused on in this study to generate 14 datasets containing VP2 nucleotide sequences, which are listed in Supplementary Tables 1a–h. The sequences were obtained from the NCBI GenBank (15). The first 10 datasets (Supplementary Tables 1a–f) were used for determining the diversity of VP2 protein at multiple taxonomic hierarchies (serotypes, topotypes, and lineages), and the obtained divergence value was compared with the previously established cutoff value for VP1 divergence. Datasets 11–13 (Supplementary Table 1g) were prepared with polyprotein sequences of FMDV to analyze the sub-lineage-level divergence of both VP2 and VP1 as there is no previously established cutoff value for the sub-lineage-level divergence of VP1. Twenty local isolates sequenced in the current study were compiled in dataset 11 (Supplementary Table 1g). Dataset 14 (Supplementary Table 1h) was used for the determination of conserved antigenic sites in VP2 and designing of consensus VP2 protein sequence.

### Phylogenetic Analysis Based on VP2 Coding Region

Multiple sequence alignment of each dataset was performed and subsequent phylogenetic analysis was carried out in MEGA7 using ClustalW algorithm (16). Unweighted pair group method with arithmetic mean (UPGMA) trees were constructed which indicated cutoff values of VP2 divergence at multiple subgroup levels of FMDV (serotypes, topotypes, and lineages). The VP2-based cutoff divergence value was then compared with the VP1-based cutoff value of divergence reported elsewhere (17–19). The sub-lineage-level divergence was compared by creating UPGMA trees based on both VP1 and VP2 nucleotide sequences.

### Calculation of Protein Variability Index

For calculating the protein variability index of 279 VP2 sequences (Supplementary Table 1a) of FMDV serotypes O, A, and Asia1, Protein Variability Server (PVS) (20) was used. The Wu–Kabat variability coefficient, with a variability threshold of 1.0, was calculated to find out the maximum variable positions and the most conserved regions in the VP2 proteins of all three serotypes.

### Visualization of Mutations

MEGA7-based mutation analysis was performed in two levels—one is mutations in the previously reported antigenic sites [Supplementary Table 2] of FMDV VP2 protein of serotypes O, A, and Asia1 (Supplementary Table 1a). Another one is more precise—mutation in the PVS returned conserved fragments of VP2 protein of FMDV focusing on the Asian region (Supplementary Table 1h).

### Determination of Conserved Fragments and Validation of Conservancy

Based on the aim of this study, which is the assessment of the credibility of VP2 protein in serotype-independent diagnosis of FMDV, the determination of antigenic sites that are inter-serotypically conserved is a major step. A total of 360 FMDV VP2 sequences (Supplementary Table 1h), representing the isolates of Asian territory, were taken into account for finding out the conserved regions in VP2. The PVS was used for returning conserved fragments of seven or more consecutive residues, with a variability threshold of 0.5 using Shannon diversity analysis. The accuracy of the conserved fragments was validated by comparing with the results obtained from the mutation analysis. The mutation score of each position was calculated by the analysis of VP2 protein alignment in MEGA7.

### Homology Modeling

Homology modeling of VP2 protein was performed to observe whether there are any structural dissimilarities in the 3D configuration of FMDV serotypes O, A, and Asia1 in the obtained conserved regions. For this purpose, three Bangladeshi isolates were selected as a representatives of three FMDV serotypes: BAN/JA/Ma-180/2013 (accession KJ175183) as representative of serotype O, BAN/CH/Sa-304/2016 (accession MK088171) as representative of serotype A, and BAN/DH/Sa-318/2018 (accession MN722609) as representative of serotype Asia1. SWISS-MODEL server (21) was used to search suitable template protein structures and generate models of protein 3D structure in PDB format using 1qqp as a template. The stereo-chemical quality of the generated 3D structures was validated by Ramachandran plot using PROCHEK (22). The alignment and the superimposition of the 3D structures were performed in PyMOL (23).

### Antigenicity Calculation and Structural Assessment of the Conserved Sites

The conserved fragments that fall within the previously reported antigenic sites of VP2 [Supplementary Table 2] were identified, followed by the detection of their antigenicity value by vaxijen v2.0 server (24), with a threshold of 0.4. Additionally, the surface accessibility and other structural properties of the conserved antigenic sites were determined using NetSurfP-2.0 server (25), which provides information regarding protein secondary structure, probability of disordered residue, and the relative surface accessibility of individual amino acid residues in the protein structure.

### Designing of Consensus VP2 Protein and Mapping of Possible B Cell Epitopes

N-terminal 130 residues of VP2 were targeted for designing a consensus sequence since this region contained all the surface-exposed conserved antigenic sites of VP2. The alignment of 360 VP2 sequences (Supplementary Table 1h) was analyzed to find out the most frequently observed residue at each position in the alignment, and by combining these residues, a consensus VP2 protein sequence of 130 amino acids in length was designed. The secondary structure and surface accessibility of the designed protein was studied using NetSurP-2.0 server, and a 3D model was generated using SWISS-MODEL server, followed by visualizing at PyMOL. The possible B cell epitopes were identified using three of the most popular epitope prediction tools [BepiPred 1.0 (26), ABCpred, and Bcepred (27)] to minimize false-positive predictions. The predicted epitopes from each server were compared manually, and the common epitopes were chosen as the most probable B cell epitopes.

## Results

### Evolutionary Divergence of VP2 in Comparison with VP1

We determined the VP2-based cutoff divergence value of FMDV at serotype, topotype, lineage, and sub-lineage levels using a UPGMA phylogenetic study. With these values, a comparative analysis between VP2- and VP1-based divergence has been shown (Table 1).

**Table 1:** Comparative evolutionary divergence of foot-and-mouth disease virus (FMDV) at multiple taxonomic levels based on VP2 and VP1.

**Serotype-level divergence**							
**Serotype**		**O**		**A**		**Asia1**	
VP2-based nucleotide divergence value		14%							
VP1-based nucleotide divergence value		30–50% (17)							
**Topotype-level divergence**							
**Serotype**		**O**		**A**		**Asia1**	
Topotype		ME-SA, SEA, Euro-SA, CATHAY, EA-1, EA-2, EA-3, WA		ASIA, AFRICA, Euro-SA		ASIA	
VP2-based nucleotide divergence value		5.5%		8%		–	
VP1-based nucleotide divergence value		15–20% (18)							
**Lineage-level divergence**							
**Serotype**	**O**			**A**			**Asia1**
**Topotype**	**ME-SA**	**SEA**	**Euro-SA**	**ASIA**	**AFRICA**	**Euro-SA**	**ASIA**
Lineage	Ind2001 PanAsia I PanAsia II	CAM-94 MYA-98	O1 O2	G-VII A-15 A22 Iran-05 Iran-87 Iran-96 Thai-87 Sea-97	G-I G-II G-III G-IV G-V G-VI G-VII	A5 A12 A24 A81	G-I G-II G-III G-IV G-V G-VI G-VII G-VIII G-IX
VP2-based nucleotide divergence value	2.9%	5.5%	4.25%	3.9%	5.4%.	5.1%	4.8%
VP1-based nucleotide divergence value	7.5% (19)						
**Sub-lineage-level divergence**							
**Lineage**		**O/ME-SA/Ind2001**		**O/ME-SA/PanAsia II**		**A/ASIA/IRN-05**	
Sub-lineage		Ind2001d Ind2001BD1 Ind2001BD2		ANT-10 FAR-09 PUN-10		BAR-08 AFG-07 ARD-07	
VP2-based nucleotide divergence value		2.9%		1.8%		2.7%	
VP1-based nucleotide divergence value		3.5%		2.7%		2.7%	

Thirteen individual datasets (Supplementary Tables 1a–g) were used to generate unweighted pair group method with arithmetic mean trees for comparing the VP2- and VP1-based cutoff divergence values of different subgroups under three serotypes of FMDV(O,A, and Asia1).

### Serotype-Level Divergence

Serotype-level divergence analysis performed considering the VP2 sequences of 279 FMDV isolates (Supplementary Table 1a) representing serotypes O, A, and Asia1 revealed two closest clusters (serotypes O and Asia1) in the tree [Supplementary Figure 1], providing a cutoff value of ~14% divergence among these three FMDV serotypes. In contrast, the serotypes of FMDV possess at least 30% nucleotide sequence diversity in the VP1 coding region (17). These findings support that VP2-based nucleotide divergence ensures higher conservancy than VP1 at the serotype level.

### Topotype-Level Divergence

UPGMA phylogenetic analysis of eight topotypes of FMDV serotype O and three topotypes of serotype A using datasets 2 and 3 (Supplementary Tables 1b,c) showed 5.5 and 8% cutoff divergence values, respectively (Supplementary Figures 2A,B). In contrast, the topotypes exhibit ≥15% variation based on the VP1 coding sequence (17, 19).

### Lineage-Level Divergence

The topotypes of FMDV are subdivided into different lineages based on at least 7.5% VP1 nucleotide divergence. Here we measured 2.9% inter-lineage VP2 sequence variation between PanAsia-I and PanAsia-II of the most dominant ME-SA topotype in the Indian subcontinent. Topotype South East Asia (SEA) of serotype O showed 5.5% inter-lineage divergence between two lineages (CAM-94 and MYA-98) and O/Euro-SA provided a cutoff value of 4.25% divergence between O1 and O2 lineages (Supplementary Figures 3A–C). The lineages of A/Euro-SA topotype (A5, A12, A24, and A81) provided a cutoff value of 5.1% divergence (Supplementary Figure 4C). Among eight different lineages of A**/**ASIA topotype, Thai-87 and Sea-97 were found to be the most closely related and provided a cutoff value of 3.9% divergence. The African topotype of serotype A showed 5.4% inter-lineage divergence (Supplementary Figures 4A,B). The only one topotype of serotype Asia1 (topotype ASIA) showed 4.8% inter-lineage divergence between two closest lineages, G-VIII and G-IX [Supplementary Figure 5]. Moreover, we observed that topotypes from the Asian region showed less inter-lineage diversity in comparison with the African or the European and South American topotypes.

### Sub-lineage-Level Divergence

Sub-lineage-level divergence analysis among three sub-lineages of O/ME-SA/Ind2001 lineage showed VP2 to be less divergent than VP1 (Supplementary Figures 6A,B). In VP1-based phylogeny, Ind2001BD1 and Ind2001BD2 were found to be the closest sub-lineages (3.5% nucleotide divergence). In contrast, the VP2-based phylogeny showed Ind2001d and Ind2001BD2 to be the closest relatives (2.9% nucleotide divergence). Similarly, sub-lineages of PanAsia II showed less divergence in VP2 (1.8% nucleotide divergence) than VP1 (2.7% nucleotide divergence). PUN-10 and FAR-09 were found to be the closest sub-lineages in the case of VP1, while these were ANT-10 and FAR-09 in the case of VP2 (Supplementary Figures 7A,B). The sub-lineages of IRN-05 showed equal divergence for both VP1 and VP2 (2.7% nucleotide divergence), where BAR-08 and AFG-07 were found to be the closest groups in each case (Supplementary Figures 8A,B). Overall, the phylogenetic analysis ensured a higher conservancy of VP2 over VP1 at each subgroup level.

### Protein Variability Index and Mutational Frequency

**Figure 1:**
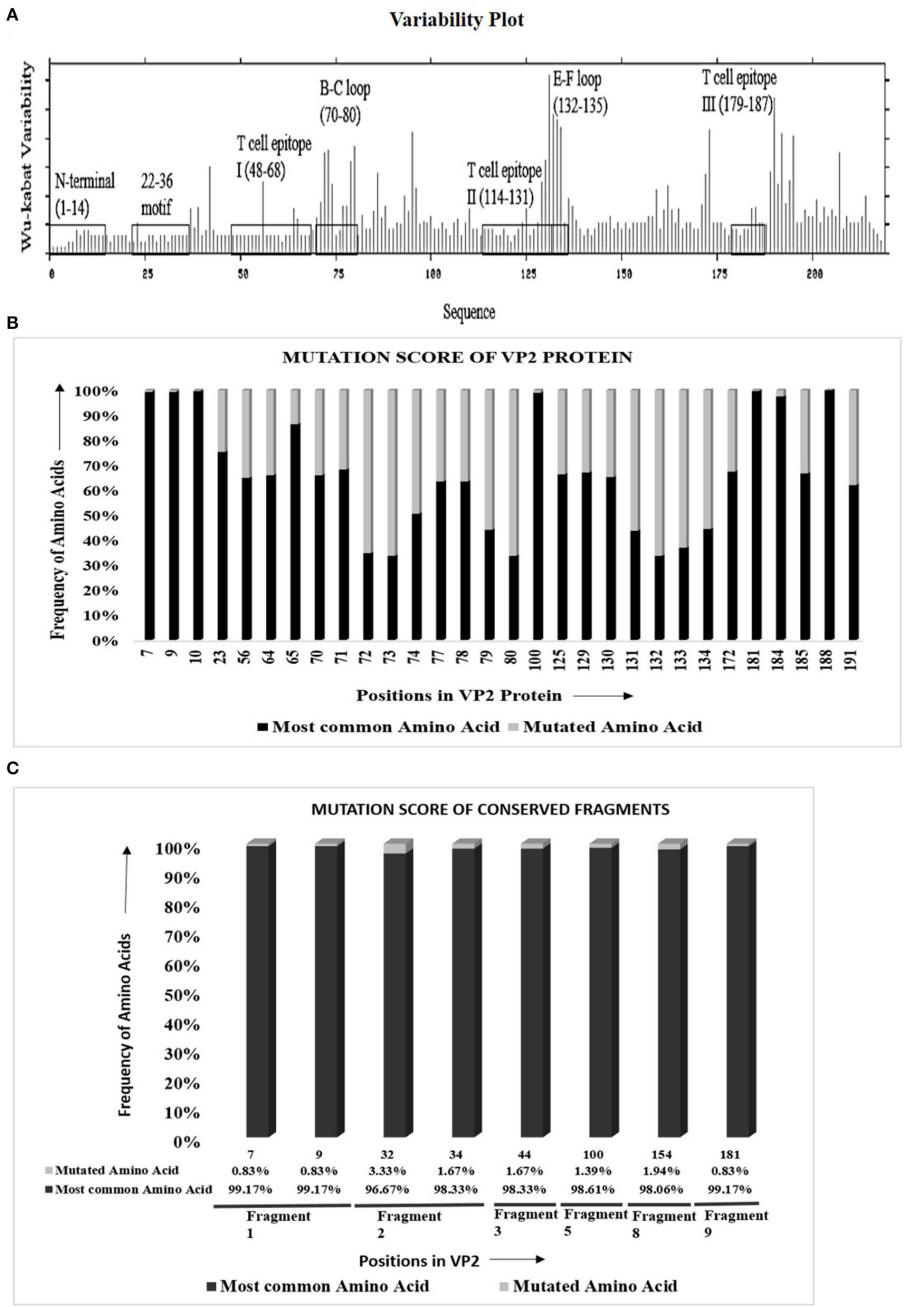
Visualization of amino acid divergence in VP2 protein of foot-and-mouth disease virus serotypes O, A, and Asia1. **(A)** Wu–Kabat protein variability plot for VP2 protein using Protein Variability Server (PVS). The plot was generated using the protein alignment of 279 VP2 sequences. The marked regions indicate the previously reported antigenic sites of VP2. The X and the Y axis of the plot, respectively, delineates the amino acid positions of VP2 and Wu–Kabat variability coefficient. **(B)** Graphical representation for the overall mutation score of the mutated sites in VP2. The protein alignment of 279 VP2 sequences was used to carry out the mutational study. For each position, the frequency of most common and mutated amino acids is represented with black and gray color, respectively. The X axis shows the amino acid position of VP2, and the Y axis shows the frequency of mutated and most common amino acids. Only the previously reported antigenic sites were taken into consideration for the mutational study. **(C)** Graphical representation for overall mutational score of the mutated positions in the conserved fragments of VP2 protein. The protein alignment of 360 Asian VP2 sequences was used to identify conserved fragments of VP2 using PVS and mutational study of the fragments. The X axis shows the amino acid position of VP2, and the Y axis shows the frequency of mutated and most common amino acids. For each position, the most common amino acids are presented with black color, and the other least common amino acids are presented with gray color. The percentage of frequency of amino acids is attached down to the respective positions. The respective conserved fragments to which the amino acid positions belong are mentioned as fragment numbers (positions 7 and 9 in fragment 1; 32 and 34 in fragment 2; and 44, 100, 154, and 181 in fragments 3, 5, 8, and 9, respectively). Only the previously reported antigenic sites were taken into consideration for the mutational study.

The protein variability analysis and mutational study using 279 VP2 sequences (Supplementary Table 1a) showed that 147 of 218 (67.4%) amino acids were inter-serotypically conserved. In the Wu–Kabat plot (Figure 1A), the N-terminal end and the 22–36 motif were found to be highly conserved. A higher Wu–Kabat variability coefficient was found in the B–C and E–F loops and near the C-terminal end.

Mutational study of the previously reported antigenic regions of VP2 delineated that, among 71 varied sites in VP2, only 29 fall within the antigenic regions positioned at three sites each in the N-terminal, T cell epitope I, E–F loop, and T cell epitope III, nine in the B–C loop, and four in T cell epitope II [Supplementary Table 3]. In the antigenic regions, only 25 sites were detected to be occupied by more than two amino acids, and as expected, significant variations were observed in the B–C loop and the E–F loop.

The mutational score calculation (Figure 1B) of the mutated sites revealed a higher frequency of most common amino acids in comparison to mutated amino acids. Three mutated sites of the N-terminal of VP2 (positions 7, 9, and 10) provided more than 99% frequency of the most common amino acids in comparison with the mutated ones. The only mutated position of the 22–36 motif is position 23, which showed 75.27% frequency of the most common amino acid, threonine, which is satisfactorily high. Among the three T cell epitopes, a high percentage of mutational frequency was observed in T cell epitope II that showed a mutation at four positions, with the mutation score ranging from 32.98% to 56.29%. The highest mutational frequency was observed in the B–C and the E–F loops, with mutation scores ranging from 31.75 to 66.31%. Critical amino acids at positions 100, 172, 188, and 191 showed 98.92, 67.38, 100, and 62% frequency of the most common amino acids, respectively. Overall, the study confirmed a remarkable inter-serotype conservancy of VP2 at the reported antigenic sites.

### Inter-serotypically Conserved Regions within VP2 Protein

VP2 sequences representing the isolates of the Asian territory (Supplementary Table 1h) revealed nine inter-serotypically conserved fragments (Table 2) after Shannon diversity analysis. The accuracy of the conservation of all nine fragments was validated by a mutational study, which also supported the inter-serotypic conservancy of the fragments, except in eight positions with only 0.83–3.33% insignificant mutational frequency (Figure 1C). Moreover, homology modeling and superimposition of VP2 proteins from three serotypes displayed no structural dissimilarities in the conserved regions and supported that the 3D configurations of the conserved regions are inter-serotypically invariant (Supplementary Figure 9A). Using the modeled 3D structures, the generated Ramachandran plot (Supplementary Figure 9B) showed that 89.4–91 and 8.5–9% of residues of the modeled structure are within the most favored (red) and the additional allowed regions (yellow), ensuring the highly reliable stereochemical properties of the generated models.

**Table 2:** Inter-serotypically conserved fragments in VP2 obtained by Protein Variability Server and their antigenicity assessment.

**Conserved fragments in VP2**	**Reported antigenic sites in VP2**	**Conserved antigenic sites in VP2**	**Sequence of the conserved antigenic sites**	**Length**	**Antigenicity value**	**Presence of antigenicity**
Fragment 1 (1–22)	N-terminal (1–14)	Site I (1–14)	DKKTEETTLLEDRI	14	0.4189	Yes
Fragment 2 (24–36)	22–36 motif	Site II (24–36)	STTQSSVGVTYGY	13	1.1031	Yes
Fragment 3 (43–55)	T cell epitope I (48–68)	Site III (48–55)	TSGLETRV	8	0.7873	Yes
Fragment 4 (57–63)	T cell epitope I (48–68)	Site IV (57–63)	QAERFFK	7	−0.8310	No
Fragment 5 (99–106)	No reported antigenic site	–	–	–	–	–
Fragment 6 (111–124)	T cell epitope II (114–132)	Site V (114–124)	NQFNGGCLLVA	11	0.7728	Yes
Fragment 7 (139–148)	No reported antigenic site	–	–	–	–	–
Fragment 8 (150–158)	No reported antigenic site	–	–	–	–	–
Fragment 9 (174–183)	T cell epitope III (179–187)	Site VI (179–183)	LVVMV	5	Too low to detect	No

*Shannon variability index with <0.5 variability was used as a threshold for finding out conserved fragments of seven or more consecutive residues. The antigenicity value of the fragments that fall in the previously reported antigenic sites of VP2 was calculated with Vaxijen v2.0 server, and fragments with antigenicity values of 0.4 or above were considered to be antigenic*.

### Antigenicity and Surface Accessibility of the Conserved Fragments

**Figure 2:**
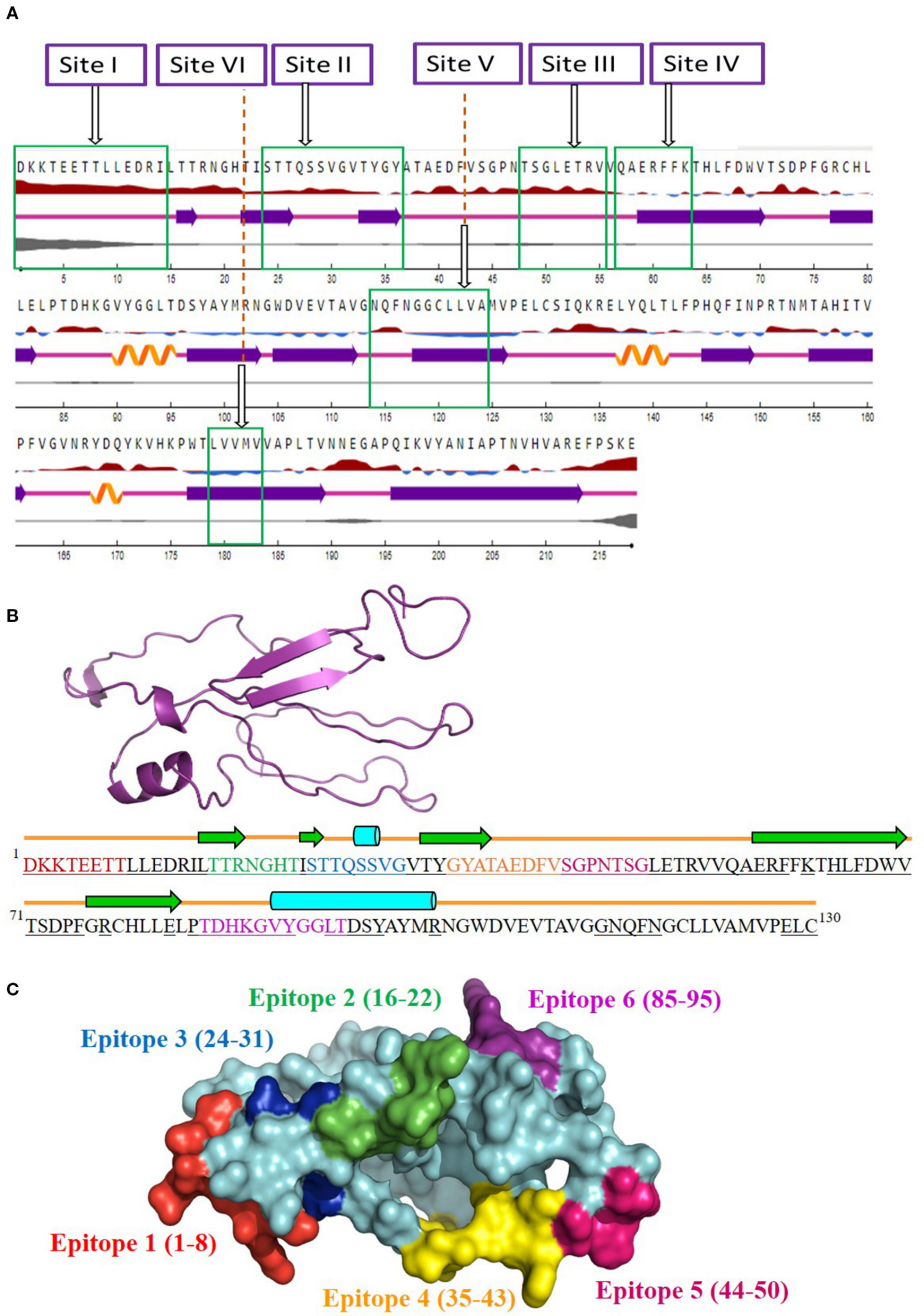
Screening of surface-accessible conserved antigenic regions in VP2 and designing of consensus VP2 sequence along with mapping of possible B cell epitopes. **(A)** Graphical representation of the surface accessibility and the structural properties of VP2 protein. Relative surface accessibility is presented in blue and red elevation and inclination (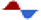), where red indicates the exposed surface and blue indicates the buried regions; threshold was kept at 25%. Curved (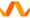), arrowed (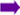), and lined (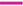) cartoons depict the secondary structure of different regions of the protein, indicating helical, stranded, and coiled structure, respectively. The disorderliness of residues in the protein is shown by gray-colored curvature, where the thickness of the gray line equals the probability of the disordered residue. **(B)** Designed consensus VP2 protein sequence and its predicted 2D and 3D models. The sequence was designed by combining the most common amino acids at positions 1–130 of VP2 protein. The green arrows highlight the predicted β-strands. Cylinders and lines, shown in cyan and orange colors, highlight the α-helices and connecting loops, respectively. The underlined amino acid residues represent the surface-accessible regions of the designed protein. The 3D structure for the designed protein, in ribbon format, is shown as an insert in the upper-left corner of the figure. Mapped B cell epitopes have been indicated with separate colors. **(C)** Predicted B cell epitopes mapped onto the 3D structure of the designed protein. The whole protein has been colored in cyan, whereas epitopes 1–6 are represented in red, green, blue, orange, pink, and violet, respectively.

The antigenicity, surface accessibility, and other structural properties of conserved fragments were determined to validate their credibility as suitable candidates for serotype-independent detection of FMDV. Among the nine conserved fragments of VP2, six fell within the previously reported antigenic sites (fragments 1–4, 6, and 9), whereas non-antigenic sites comprised three other fragments (5, 7, and 8). Comparing the PVS returned conserved fragments and the previously reported antigenic sites, we selected six conserved antigenic sites (sites I–VI) in VP2 for calculating the antigenicity value. Among these six sites, four (sites I, II, III, and V) were found to be antigenic, with a sufficient antigenicity value ranging from 0.4189 to 1.1031 (Table 2). Among these four conserved antigenic sites, three were found to be entirely surface-exposed (sites I, II, and III), and site V showed partial surface accessibility with a threshold of 25% (Figure 2A). In site V (114–124), the first three amino acids (N, Q, and F) were found to be surface-exposed, providing the antigenicity value of 0.7728 (Table 2), whereas the remaining amino acids of this site were buried. In contrast, site IV had only two surface-exposed amino acids, and site VI was completely buried. Sites I and III showed a coiled structure, although there is a probability of significant-level disorderliness in site I. The other sites have not shown any significant disordered residue. Sites II, IV, and V showed a co-abundance of stranded and coiled structures, and site VI falls in an entirely stranded structured region (Figure 2A).

### Designed VP2 Protein and Mapped B Cell Epitopes

Since the four surface-exposed conserved antigenic sites, sites I, II, III, and V (Figure 2A), are located within the N-terminal 130 residues of VP2, a consensus VP2 protein was designed by combining the first 130 most common amino acids of VP2 sequentially. The designed protein (Figure 2B) showed an abundance of coiled loop structures, which indicate their possible role in the antigenicity of VP2. Five β-strands and two α-helices were also observed. N-terminal 75 residues were found to be exclusively surface-exposed, which have been indicated by underlined residues in Figure 2B. Six B cell epitopes have been mapped in the designed protein, which are shown in Figures 2B,C. The epitopes are ^1^DKKTEETT^8^, ^16^TTRNGHT^22^, ^24^STTQSSVG^31^, ^35^GYATAEDFV^43^, ^44^SGPNTSG^50^, and ^85^TDHKGVYGGLT^95^. All the epitopes were found to be surface-exposed (Figure 2B). A secondary structure analysis of the epitopes revealed that all the epitopes form part of the coiled connecting loops; also, epitopes 1 and 5 entirely fall in the looped region. β-sheeted regions were observed in epitope 2 [first three amino acids (aa)], epitope 3 (first two aa), and epitope 4 (first four aa). Helices were observed in epitopes 3 and 6. Residues near the C-terminal of the designed protein were found to be buried under the surface structure (Figure 2B).

## Discussion

Foot-and-mouth disease is a major threat to an economically important livestock population and caused by the foot-and-mouth disease virus (28). Being an RNA virus, FMDV lacks the proofreading mechanism during virus replication resulting in the extensive genetic heterogeneity of the virus. High genetic diversity results in the differentiation of the viruses into different serotypes, topotypes, lineages, and sub-lineages (11, 29). For selecting an appropriate vaccine strain, determination of the infecting FMDV serotype is important during FMD diagnosis. Even so, serotype-independent detection is the most preferred method for the rapid checking of animals during emergency outbreak or international animal trade. Because the detection of individual serotype needs separate diagnostic kits, which eventually increases cost and diagnosis time. Although non-structural protein (NSP)-based diagnostic approaches can offer serotype-independent detection, the generation of anti-NSP antibody requires more time than anti-structural protein (SP) antibodies (30), thus limiting their ability to be used in early diagnosis of the disease. Early diagnosis of the infection will allow the breeder to rapidly separate the infected animals from the uninfected ones, consequently terminating the spread of the disease to the entire farm during an outbreak. Thus, SP-based serotype-independent detection protocols are more beneficial over NSP-based protocols.

Among the four structural proteins of FMDV, VP4 is completely internalized (31) and thus cannot be used for the development of any diagnostic approach. Although VP1-based diagnostic methods are widely used (32–35), serotypic structural diversity due to VP1 sequence variation can be responsible for the false-negative identification of anti-viral antibody and limiting serotype-independent detection of FMD. Considering robust diversity within the VP1 coding sequence, another surface protein having less diversity and potential immunogenicity should be targeted for the serotype-independent detection of anti-FMDV antibody (36). Since VP2 protein is reported to be less divergent than VP1 and VP3 (10–12) and it contains humoral response inducing surface epitopes, using VP2 is more reliable in developing a serotype-independent diagnostic tool. Therefore, we hypothesized that VP2 protein can offer a solution towards serotype-independent diagnosis of FMD. To validate this hypothesis, we at first analyzed the evolutionary divergence of VP2 in comparison with VP1. Besides, we identified the surface-exposed conserved antigenic regions in VP2, and based on the findings, we designed a consensus protein that can be used for the development of a serotype-independent FMD diagnostic kit. Since our prime focus was the Asian region, we used only the most prevalent serotypes (O, A, and Asia1) circulating in this region.

Firstly, VP2-based evolutionary divergence analysis ensured a remarkably higher conservancy in VP2 than VP1 at each taxonomic level (serotypes, topotypes, lineages, and sub-lineages). These findings corroborated with other previous studies stating that VP2 is more conserved than VP1 and VP3 at the serotype level (10–12). However, none of the previous studies showed the extent of VP2-based evolutionary divergence at the other hierarchical levels that we demonstrated here. For instance, Carrillo et al. performed the comparative genomic analysis of 103 FMDV isolates representing all seven serotypes and reported VP2 protein (47% invariant aa) to be more conserved than VP1 (24% invariant aa). Also, transitions *vs*. transversions (Ts/Tv) rate and synonymous *vs*. non-synonymous mutation rate (Syn/non-syn) were higher in VP2 than in VP1 (10). Moreover, Chitray et al. found 33% and 54.2% variant aa in VP2 and VP1, respectively, after a comparative study of 53 sequences of serotype A and O where the Ts/Tv and Syn/non-syn rates were also higher in VP2 (11). After a comparative analysis of 35 sequences of all serotypes, Feng et al. also found VP2 (70–97% aa sequence similarity) to be more conserved than VP1 (45–96% aa sequence similarity) (12). Herein we found 67.4% invariant aa in VP2 after a mutational study of 279 sequences of three FMDV serotypes circulating in Asia (O, A, and Asia1). Although the previous studies relate to our study regarding VP2 conservancy, we extended our analysis by determining the VP2-based cutoff divergence value at each subgroup level of three dominantly circulating Asian serotypes of FMDV by UPGMA phylogenetic analysis using 13 individual datasets (datasets 1–13/Supplementary Tables 1a–g), while the previous others showed only the serotype-dependent distinction of clusters by neighbor-joining phylogeny using a rather small dataset. Nevertheless, other studies along with our findings conclude that VP2 is significantly conserved than VP1. Thus, any diagnostic tool designed based on VP2 protein will offer a relatively more stable diagnosis approach.

Several previous studies (37, 38) proposed the N-terminal end of VP2 to be inter-serotypically conserved, which we showed in our study by multiple bioinformatics analysis (protein variability analysis, mutational study, and identification of B cell epitopes). Salem et al. designed and developed an indirect ELISA using the N-terminal conserved regions of VP2, which provided higher sensitivity than VNT and LPBE (37). However, that study used one Egyptian SAT2 isolate (gb|AAZ83686) as model for the development of a VP2-based type-independent indirect ELISA approach and used only the O, A, and SAT2 antisera to evaluate the sensitivity and the specificity of their kit. The authors took 64 sequences for the VP2 invariant site prediction and phylogeny reconstruction (11 O, 10 Asia1, 11 A, and the rest were of SAT1–3 serotypes). We believe that this small dataset does not ensure the conservation of VP2 among the highly diverse O, A and Asia1 serotypes prevailing in the Asian region, and thus using their developed kit should not offer a better output for this region. Freiberg et al. reported another interesting study showing a type-independent detection of foot-and-mouth disease virus by monoclonal antibodies (mAbs) raised against FMDV A22 Iraq/1964, Asia1 Shamir Israel/1989, and SAT1 Zimbabwe/1989 (38). They showed that the monoclonal antibodies bind to the N-terminal of VP2 and were able to recognize 27 representative isolates of six serotypes (O, A, Asia1, C, and SAT1-2). Before concluding the applicability of their recommended mAbs for type-independent detection of FMDVs in the whole Asian region, *in silico* study should be performed to ensure the conservancy of the utilized isolates with other vast majority of isolates circulating in Asia. Oem et al. developed another ELISA-based detection strategy that uses the recombinant pentameric subunit of FMDV as a diagnostic tool, but they used only FMDV type O kit for their study (39). None of those studies offer any solution for the development of diagnostic kits in the Asian region where serotypes O, A, and Asia1 are the most prevalent types. In our study, we determined VP2-based cutoff divergence values (Table 1) at multiple taxonomic levels using 13 individual datasets carrying the sequences of almost all subgroups of FMDV circulating in Asia, which will enable the scientific community to visualize the actual picture of VP2 divergence in FMDV strains circulating in the whole Asian region. By establishing the conservation level of VP2 for all Asian subgroups, we demonstrated the possibility of using VP2 for type-independent diagnosis of FMDV in the whole Asian region, which is also a premier report globally.

Interestingly, we observed a difference in divergence among lineages belonging to the same serotype. O/SEA topotype showed the highest inter-lineage variation (with 5.5% divergence) in the VP2 region, while the lowest inter-lineage divergence was observed for O/ME-SA topotype (2.9%). Similarly, inter-lineage divergence in the VP2 region of African (5.4%) and Asian (3.9%) topotypes was the highest and the lowest lineage level divergence for serotype A. This difference in divergence among lineages within the same serotype may possibly be due to the recombination events in FMDVs within the conserved region of VP2 as described by Jamal et al. (40, 41). Importantly, at each hierarchical level, the divergence values for VP2 were lower than those for VP1, except the sub-lineage-level divergence of A/IRN-05 lineage where the values were equal.

Another noteworthy finding of the study is the determination of inter-serotypically conserved fragments in VP2. The highly antigenic, surface-exposed, and conserved fragments can be efficiently used for a serotype-independent diagnosis of FMD. The protein variability data revealed new highly conserved regions in VP2. Nine previously undescribed inter-serotypically invariant fragments within VP2 have been identified in this study (Table 2). The antigenicity value calculation determined four sites to have a higher antigenicity value than the preset threshold. The sites are DKKTEETTLLEDRI (1–14), STTQSSVGVTYGY (24–36), TSGLETRV (48–55), and NQFNGGCLLVA (114–124) (Table 2). The sites were found to have surface exposure and a functionally active structural configuration (Figure 2A), indicating their possible interaction with the immune cells. Thus, these four sites can be a promising focus in designing a serotype-independent diagnostic approach for FMDV detection. Considering this promise, we designed a consensus VP2 protein by combining the first 130 most common amino acids of VP2 sequentially and identified six possible B cell epitopes in this protein. Also, the epitopes were found to be surface-exposed and have a functionally active structural configuration. These findings suggest that the designed protein can be a suitable candidate for the development of a serotype-independent diagnostic tool using ELISA-based approaches. Similar approaches were developed by Salem et al. using one Egyptian SAT2 isolate of FMDV (gb|AAZ83686), which showed the expected level of sensitivity in FMD detection (37). No such strategy is available in the Asian region until now. Yang et al. described a major epitope in VP2 (^8^TLLEDRILT^16^) (42), showing that this linear epitope was highly conserved among 21 isolates of all seven serotypes of FMDV and provided proof of concept on an effective serotype-independent test by developing monoclonal antibodies directed to that peptide. Although our study lacks such proof of concept, we performed a robust divergence analysis using a lot more sequences than the previous one. Thus, we can claim our designed protein to be much more reliable at the question of developing a kit for the whole Asian region. Whereas previous studies showed experimental data, we focused on analyzing the actual divergence pattern of VP2 among all Asian subgroups (except a few from which no VP2 sequences were available), and thus proposed a designed protein that will be inter-serotypically highly conserved among all Asian strains. In agreement, our proposed protein will contribute to the scientific world by offering a similar output for all Asian isolates, which was not claimed by previous studies. Though we have lack of experimental data supporting our conclusions, the robustness of our data analysis will widen the window for developing a type-independent detection strategy for FMDV as we are providing necessary information regarding the conserved epitopes of a designed VP2 protein along with their surface accessibility and structural assessment. Finally, the future direction of our study will be the development of a VP2-based indirect ELISA for FMD diagnosis using the designed protein. The first following step in this purpose will be the synthesis of the designed VP2 protein, and then the protein will be used as a coating antigen in an indirect ELISA approach for type-independent detection of anti-FMDV antibody.

## Conclusions

The study has validated that the VP2 protein of FMDV is significantly conserved among three FMDV serotypes (O, A, and Asia1) and other hierarchical subgroups in comparison with VP1. The study also reported four previously undescribed, surface-exposed, inter-serotypically conserved antigenic sites in VP2. Based on these findings, the study reported a designed consensus VP2 protein that carries promise to be used in the development of a serotype-independent diagnostic tool, which will be applicable to the whole Asian region. This is the first report showing the overall diversity of VP2 protein where we validated the credibility of VP2 in serotype-independent detection of FMDV.

## Data Availability Statement

The datasets presented in this study can be found in online repositories. The names of the repository/repositories and accession number(s) can be found in the article/Supplementary Material.

## Author Contributions

MS designed the study. IM performed the experiments, analyzed the results, and wrote the manuscript. SA and AA contributed to the analysis and helped in writing the manuscript. MS and MH provided technical support. All authors reviewed and approved the final manuscript.

## Conflict of Interest

The authors declare that the research was conducted in the absence of any commercial or financial relationships that could be construed as a potential conflict of interest.

## References

[1] Alam SS Amin R Rahman MZ Hossain MA Sultana M. Antigenic heterogeneity of capsid protein VP1 in foot-and-mouth disease virus (FMDV) serotype Asia 1. Adv App Bioinform Chem. (2013) 6:37. 10.2147/AABC.S49587 23983476 PMC3751384

[2] Siddique MA Ullah H Nandi SP Chakma D Sultana M Hossain MA. Molecular characterization of foot-and-mouth disease virus type O from wild pig in Bangladesh. Bangladesh J Microbiol. (2014) 31:41–5. 10.3329/bjm.v31i1.2846427789636

[3] Knight-Jones T Rushton J. The economic impacts of foot and mouth disease–what are they, how big are they and where do they occur? Prevent Vet Med. (2013) 112:161–73. 10.1016/j.prevetmed.2013.07.013 23958457 PMC3989032

[4] Ullah H Siddique MA Sultana M Hossain MA. Complete genome sequence of foot-and-mouth disease virus type A circulating in Bangladesh. Genome Announc. (2014) 2:e00506–14. 10.1128/genomeA.00506-14 24926048 PMC4056291

[5] Sultana M Siddique MA Momtaz S Rahman A Ullah H Nandi SP. Complete genome sequence of foot-and-mouth disease virus serotype O isolated from Bangladesh. Genome Announc. (2014) 2:e01253–13. 10.1128/genomeA.01253-13 24503997 PMC3916491

[6] Ali MR Ullah H Siddique MA Sultana M Hossain MA. Complete genome sequence of pig-originated foot-and-mouth disease virus serotype O from Bangladesh. Genome Announc. (2016) 4:e01150–16. 10.1128/genomeA.01150-16 27789636 PMC5084860

[7] Marquardt O Adam K-H. Foot-and-mouth disease virus subtyping by sequencing VP1 genes. Vet Microbiol. (1990) 23:175–83. 10.1016/0378-1135(90)90147-N 2169671

[8] Mohapatra JK Sanyal A Hemadri D Tosh C Sabarinath G Venkataramanan R. Sequence and phylogenetic analysis of the L and VP1 genes of foot-and-mouth disease virus serotype Asia1. Virus Res. (2002) 87:107–18. 10.1016/S0168-1702(02)00006-0 12191774

[9] Samuel AR Knowles NJ. Foot-and-mouth disease type O viruses exhibit genetically and geographically distinct evolutionary lineages (topotypes). J Gen Virol. (2001) 82:609–21. 10.1099/0022-1317-82-3-609 11172103

[10] Carrillo C Tulman E Delhon G Lu Z Carreno A Vagnozzi A. Comparative genomics of foot-and-mouth disease virus. J Virol. (2005) 79:6487–504. 10.1128/JVI.79.10.6487-6504.2005 15858032 PMC1091679

[11] Chitray M De Beer TAP Vosloo W Maree FF. Genetic heterogeneity in the leader and P1-coding regions of foot-and-mouth disease virus serotypes A and O in Africa. Arch Virol. (2014) 159:947–61. 10.1007/s00705-013-1838-9 24221247 PMC4010724

[12] Feng Q Yu H Liu Y He C Hu J Sang H. Genome comparison of a novel foot-and-mouth disease virus with other FMDV strains. Biochem Biophys Res Commun. (2004) 323:254–63. 10.1016/j.bbrc.2004.08.086 15351730

[13] Anasir MI Poh CL. Advances in antigenic peptide-based vaccine and neutralizing antibodies against viruses causing hand, foot, and mouth disease. Int J Mol Sci. (2019) 20:1256. 10.3390/ijms20061256 30871133 PMC6471744

[14] Swindell SR Plasterer TN Seqman, in Sequence Data Analysis Guidebook. Totowa, NJ: Springer (1997). p. 75–89.

[15] Benson DA Cavanaugh M Clark K Karsch-Mizrachi I Lipman DJ Ostell J GenBank. Nucleic Acids Res. (2012) 41:D36–42. 10.1093/nar/gks1195 23193287 PMC3531190

[16] Thompson JD Higgins DG Gibson TJ. CLUSTAL W: improving the sensitivity of progressive multiple sequence alignment through sequence weighting, position-specific gap penalties and weight matrix choice. Nucleic Acids Res. (1994) 22:4673–80. 10.1093/nar/22.22.4673 7984417 PMC308517

[17] Knowles N Samuel A. Molecular epidemiology of foot-and-mouth disease virus. Virus Res. (2003) 91:65–80. 10.1016/S0168-1702(02)00260-5 12527438

[18] Samuel AR Knowles NJ. Foot-and-mouth disease virus: cause of the recent crisis for the UK livestock industry. Trends Genet. (2001) 17:421–4. 10.1016/S0168-9525(01)02374-5 11485797

[19] Siddique M Ali M Alam A Ullah H Rahman A Chakrabarty R. Emergence of two novel sublineages Ind2001 BD 1 and Ind2001 BD 2 of foot-and-mouth disease virus serotype O in Bangladesh. Transbound Emerg Dis. (2018) 65:1009–23. 10.1111/tbed.12834 29457368

[20] Garcia-Boronat M Diez-Rivero CM Reinherz EL Reche PA. PVS: a web server for protein sequence variability analysis tuned to facilitate conserved epitope discovery. Nucleic Acids Res. (2008) 36(Suppl. 2):W35–41. 10.1093/nar/gkn211 18442995 PMC2447719

[21] Schwede T Kopp J Guex N Peitsch MC. SWISS-MODEL: an automated protein homology-modeling server. Nucleic Acids Res. (2003) 31:3381–5. 10.1093/nar/gkg520 12824332 PMC168927

[22] Laskowski RA MacArthur MW Moss DS Thornton JM PROCHECK: a program to check the stereochemical quality of protein structures. J Appl Crystallograp. (1993) 26:283–91. 10.1107/S0021889892009944

[23] Schrodinger L The PyMOL Molecular Graphics System. (2010) 1:0.

[24] Doytchinova IA Flower DR. VaxiJen: a server for prediction of protective antigens, tumour antigens and subunit vaccines. BMC Bioinform. (2007) 8:4. 10.1186/1471-2105-8-4 17207271 PMC1780059

[25] Klausen MS Jespersen MC Nielsen H Jensen KK Jurtz VI Soenderby CK. NetSurfP-2.0: improved prediction of protein structural features by integrated deep learning. Proteins. (2019) 87:520–7. 10.1002/prot.25674 30785653

[26] Larsen JEP Lund O Nielsen M. Improved method for predicting linear B-cell epitopes. Immun Res. (2006) 2:2. 10.1186/1745-7580-2-2 16635264 PMC1479323

[27] Saha S Raghava GPS BcePred: prediction of continuous B-cell epitopes in antigenic sequences using physico-chemical properties. In: International Conference on Artificial Immune Systems. Springer (2004).

[28] Ullah H Siddique M Al Amin M Das B Sultana M Hossain M. Re-emergence of circulatory foot-and-mouth disease virus serotypes Asia1 in Bangladesh and VP1 protein heterogeneity with vaccine strain IND 63/72. Lett Appl Microbiol. (2015) 60:168–73. 10.1111/lam.12354 25370946

[29] Ali MR Alam ARU Al Amin M Ullah H Siddique MA Momtaz S. Complete genome sequence of the circulatory foot-and-mouth disease virus serotype Asia1 in Bangladesh. Genome Announc. (2017) 5:e01135–17. 10.1128/genomeA.01135-17 29074654 PMC5658492

[30] Lu Z Cao Y Guo J Qi S Li D Zhang Q. Development and validation of a 3ABC indirect ELISA for differentiation of foot-and-mouth disease virus infected from vaccinated animals. Vet Microbiol. (2007) 125:157–69. 10.1016/j.vetmic.2007.05.017 17601688

[31] Momtaz S Rahman A Sultana M Hossain MA. Evolutionary analysis prediction of peptide vaccine candidates for foot-and-mouth-disease virus types A O in Bangladesh. Evolution Bioinform. (2014) 10:EBO. 10.4137/EBO.S17027 25452681 PMC4219755

[32] Wang G Du J Cong G Shao J Lin T Xue H. Establishment of indirect ELISA diagnose based on the VP1 structural protein of foot-and-mouth disease virus (FMDV) in pigs. Sheng Wu Gong Cheng Xue Bao = Chin J Biotechnol. (2007) 23:961–6. Available online at: https://europepmc.org/article/med/18051883 18051883

[33] Chen L Fu W Hu X-J Xiong Y Pan Q Liu Q. Establishment of indirect ELISA diagnose based on the VP1 structural protein for detecting specific antibodies against foot-and-mouth disease virus in pigs. Hubei Agri Sci. (2008) 34. Available online at: http://en.cnki.com.cn/Article_en/CJFDTotal-HBNY200811034.htm18051883

[34] Stram Y Molad T Chai D Gelman B Yadin H. Detection and subtyping of foot-and-mouth disease virus in infected cattle by polymerase chain reaction and amplified VP1 sequencing. J Vet Diagnos Investig. (1995) 7:52–5. 10.1177/104063879500700107 7779964

[35] Wong CL Sieo CC Tan WS. Display of the VP1 epitope of foot-and-mouth disease virus on bacteriophage T7 and its application in diagnosis. J Virol Methods. (2013) 193:611–9. 10.1016/j.jviromet.2013.07.053 23933075

[36] Monjane A Vieira I Improvement of a Liquid Phase Blocking ELISA for Enhanced Detection and Measurement of Antibodies Against the SAT 3 Serotype of FMDV. Pretoria: University of Pretoria (2016).

[37] Salem R El-Kholy AA Omar OA Abu el-naga MN Ibrahim M Osman G. Construction, expression and evaluation of recombinant VP2 protein for serotype-independent detection of FMDV seropositive animals in Egypt. Sci Rep. (2019) 9:10135. 10.1038/s41598-019-46596-9 31300744 PMC6626030

[38] Freiberg B Höhlich B Haas B Saalmüller A Pfaff E Marquardt O. Type-independent detection of foot-and-mouth disease virus by monoclonal antibodies that bind to amino-terminal residues of capsid protein VP2. J Virol Methods. (2001) 92:199–205. 10.1016/S0166-0934(00)00287-1 11226567

[39] Oem J-K Park J-H Lee K-N Kim Y-J Kye S-J Park J-Y. Characterization of recombinant foot-and-mouth disease virus pentamer-like structures expressed by baculovirus their use as diagnostic antigens in a blocking ELISA. Vaccine. (2007) 25:4112–21. 10.1016/j.vaccine.2006.08.046 17386963

[40] Jamal SM Ferrari G Ahmed S Normann P Belsham GJ. Molecular characterization of serotype Asia-1 foot-and-mouth disease viruses in Pakistan and Afghanistan; emergence of a new genetic group and evidence for a novel recombinant virus. Infect Genet Evolu. (2011) 11:2049–62. 10.1016/j.meegid.2011.09.015 21983559

[41] Jamal SM Nazem Shirazi MH Ozyoruk F Parlak U Normann P Belsham GJ. Evidence for multiple recombination events within foot-and-mouth disease viruses circulating in West Eurasia. Transbound Emerg Dis. (2020) 67:979–93. 10.1111/tbed.13433 31758840

[42] Yang B Wang M Liu W Xu Z Wang H Yang D. Identification of a serotype-independent linear epitope of foot-and-mouth disease virus. Arch Virol. (2017) 162:3875–80. 10.1007/s00705-017-3544-5 28884236

