# Supplementary material for: In silico Evolutionary Divergence Analysis Suggests the Potentiality of Capsid Protein VP2 in Serotype-Independent Foot-and-Mouth Disease Virus Detection
Source: Front Vet Sci. 2020 Sep 25;7:592. doi: 10.3389/fvets.2020.00592 (PMC7546019; doi:10.3389/fvets.2020.00592)
Supplement: Supplementary file 1 [file Data_Sheet_1.docx]

**Supplementary Tables**

**Supplementary Table 1a: Dataset 1 for serotype level diversity analysis of VP2 protein of FMDV.** The dataset Contains 279 VP2 sequences of FMDV serotype O, A and Asia1 from different regions of the world (Countries of Asia, Africa, Europe and South America). Among them 94 belonged to serotype O, 91 belonged to serotype A and 94 belonged to serotype Asia1. The dataset focuses for analyzing serotype level diversity of VP2 from both genomic and proteomic aspect. Here, only the accession no. of the sequences are listed. (Bangladeshi sequences are marked as bold).

| **Dataset 1** | | | | | | |
| --- | --- | --- | --- | --- | --- | --- |
| **MK071699** | MG983683 | MG983725 | HQ832590 | KJ146941 | KT153367 | AY687333 |
| DQ404180 | **MG983684** | MG983726 | FJ617246 | KJ146940 | KT153355 | JQ818553 |
| EF552688 | MG983685 | MG983727 | FJ617243 | KJ146939 | KT153347 | DQ989307 |
| EF552696 | MG983686 | MG983728 | KU612846 | KJ146938 | KT153344 | AY687334 |
| FJ542365 | MG983687 | MG983729 | HQ832591 | KJ146936 | KT153338 | MF372125 |
| FJ542366 | MG983688 | MG983731 | FJ617244 | KJ146932 | KT153335 | HQ268515 |
| FJ542368 | MG983689 | MG983732 | KU612843 | KJ146928 | KT153326 | DQ989305 |
| FJ542369 | MG983690 | MG983733 | KU612845 | KJ146927 | KT153368 | JF749849 |
| FJ542370 | MG983691 | MG983734 | KU612866 | KJ146926 | KT153346 | DQ989319 |
| FJ542371 | MG983692 | MG983735 | KU612863 | KJ146921 | KT153336 | DQ989321 |
| FJ542372 | MG983693 | MG983736 | KU612865 | HQ666881 | KT153334 | KY825721 |
| HQ268526 | MG983694 | MG983737 | FJ617242 | FJ617251 | KT153325 | HQ268514 |
| JX947860 | MG983695 | MG983740 | KU612844 | FJ617247 | KT153348 | HQ268513 |
| **KF985189** | MG983696 | MG983741 | KU612833 | KU612832 | KT153343 | DQ989314 |
| KJ206908 | MG983697 | **MK088171** | KJ146920 | KU612828 | KT153365 | HQ268512 |
| KJ206909 | MG983698 | KU127247 | KU612852 | KJ146933 | KT153362 | DQ989317 |
| KJ206910 | MG983699 | KU612879 | KU612851 | HQ666878 | KT153357 | DQ989318 |
| KJ825801 | MG983700 | KU612876 | KU612855 | FJ617249 | KT153351 | HQ268516 |
| KJ825802 | MG983701 | KU612872 | KU612850 | KU612824 | KT153341 | HQ113233 |
| KJ825803 | MG983702 | KU612877 | KU612834 | HQ666887 | KT153352 | MG372731 |
| KJ825804 | MG983704 | KU612875 | HQ666886 | FJ617248 | KT153350 | MF063056 |
| KJ825805 | MG983705 | KU612869 | HQ666879 | KU612884 | KT153345 | MF063055 |
| KJ825806 | MG983706 | KU612868 | FJ617245 | KU612883 | KT153327 | MF063054 |
| KJ825807 | MG983707 | KU612867 | KU612857 | FJ617250 | JN247566 | MF063053 |
| KJ825808 | MG983708 | KU612880 | KU612856 | KU612837 | KT153366 | KY825718 |
| KJ825809 | MG983709 | KU612878 | KU612854 | **MF782478** | KT153364 | JF739177 |
| KJ831678 | MG983710 | KU612881 | KU612853 | KT153322 | KT153360 | DQ989322 |
| KJ831699 | MG983711 | KU612882 | KU612831 | KT153324 | KT153353 | DQ989320 |
| KJ831717 | MG983712 | KU612871 | HQ666880 | KT153323 | KT153333 | DQ989312 |
| KM257061 | MG983713 | KU612874 | KJ146937 | KT153321 | KT153329 | AY593800 |
| KM257063 | MG983714 | KU612848 | KU612839 | KT153342 | KT153328 | AY593798 |
| KP822945 | MG983715 | KU612842 | KU612859 | KT153363 | KT153331 | DQ989316 |
| KR149703 | MG983716 | KJ146955 | KU612858 | KT153359 | KT153330 | AY593795 |
| KR149709 | MG983717 | KJ146954 | KU612835 | KT153358 | KT153340 | AJ251478 |
| KR265074 | MG983718 | KU612862 | KU612827 | KT153356 | KT153332 | AY593797 |
| KR265076 | MG983719 | KU612847 | KU612826 | KT153354 | MF372126 | KP822943 |
| KU291242 | MG983721 | KU612861 | KJ146959 | KT153349 | DQ989308 | DQ989313 |
| **KX712091** | MG983722 | KU612840 | KM189190 | KT153339 | EF149010 | DQ989304 |
| KY234501 | MG983723 | HQ666873 | KM189184 | KT153337 | JN247565 | KU737528 |
| KY657269 | MG983724 | HQ832592 | KM189183 | KT153361 | DQ989315 |  |

**Supplementary Table 1b: Dataset 2 for topotype level diversity analysis of VP2 protein of FMDV serotype O.** Serotype O of FMDV has 11 topotypes: EAST AFRICA 1 to 4 (EA-1 to 4), SOUTHEAST ASIA (SEA), EUROPE-SOUTH AMERICA (Euro-SA), INDONESIA-1 and 2 (ISA-1 and -2), CATHAY, MIDDLE EAST-ASIA (ME-SA) and WEST AFRICA (WA). This dataset contains 140 VP2 sequences of 8 topotypes and the rest three topotypes (EA-4, ISA-1, ISA-2) were not included in this study due to the unavailability of VP2 sequences of that topotypes in GenBank. Here, only the accession no. of the sequences are listed. (Bangladeshi sequences are marked as bold).

| **Dataset 2** | | | | | | |
| --- | --- | --- | --- | --- | --- | --- |
| AJ539139 | MG983726 | MG983690 | MG983702 | KR139753 | JF749854 | KJ560297 |
| AY593824 | MG983723 | MG983732 | MG983699 | KP822947 | JF749850 | JX869186 |
| KJ831737 | MG983724 | MG983734 | MG983701 | KR265083 | KR149725 | JX869183 |
| AJ539136 | KU291242 | MG983733 | MG983718 | KY322671 | KR149723 | KJ560283 |
| AJ539138 | KJ206908 | MG983731 | MG983720 | KR265082 | JF749858 | AY593819 |
| AF506822 | KJ825803 | MG983714 | MG983730 | KJ831666 | MH053315 | AY593817 |
| AH012985 | KJ825808 | MG983712 | MG983741 | KJ831680 | KR149720 | AY317098 |
| HQ632770 | KJ825801 | MG983713 | MG983727 | HQ632769 | KP202877 | EU400597 |
| JX171679 | KJ825809 | MG983708 | MG983728 | HM625676 | EU919242 | KJ831677 |
| MG983683 | KJ825804 | MG983709 | MG983729 | HQ268522 | MH053316 | KJ415243 |
| MG983735 | KJ825805 | MG983685 | MG983719 | HM625675 | EU919247 | HQ268521 |
| MG983694 | KJ825807 | **MG983684** | MG983703 | MH053318 | AY593821 | DQ248888 |
| KJ206909 | MG983715 | MG983687 | MG983691 | FJ461345 | KJ560306 | EF175732 |
| MG983695 | MG983716 | MG983705 | MG983738 | FJ461344 | KJ560303 | HQ268518 |
| MG983696 | MG983736 | MG983688 | MG983739 | HM191257 | EU448381 | KJ415245 |
| MG983697 | MG983737 | MG983706 | MG983717 | JF749857 | KJ560308 | MG983686 |
| KJ825802 | KJ825806 | MG983692 | **MK071699** | EU919240 | KJ560307 | MG983700 |
| MG983725 | MG983711 | MG983704 | **KF985189** | KJ831668 | KJ560309 | AY593811 |
| MG983721 | MG983693 | **KX712091** | AY593828 | MH053314 | KJ560305 | KJ560300 |
| KJ206910 | MG983740 | MG983698 | AY593812 | KJ831667 | KJ560304 |  |
| MG983722 |  |  |  |  |  |  |

**Supplementary Table 1c: Dataset 3 for topotype level diversity analysis of VP2 protein of FMDV serotype A.** Serotype A is divided into three topotypes: AFRICA, ASIA and EUROPE-SOUTH AMERICA (Euro-SA).  62 sequences of this three different topotypes were retrieved to create the dataset. Here, only the accession no. of the sequences are listed. (Bangladeshi sequences are marked as bold).

| **Dataset 3** | | | | | | |
| --- | --- | --- | --- | --- | --- | --- |
| KF112925 | M10975 | MH559795 | AY593764 | HM854021 | JF749844 | HQ268508 |
| MH053306 | AY593752 | MH559789 | AY593762 | KU612816 | JF749841 | HQ268510 |
| AY593761 | KF112926 | MH559784 | KY825717 | KU612822 | KF112907 | KF112919 |
| KF112916 | AY593768 | MH559779 | AY593765 | KU612817 | KF112923 | AY593755 |
| KF112910 | MH559803 | MH746921 | HM854025 | HQ832587 | KF112922 | KF152935 |
| MH053305 | MH559787 | MH426574 | KJ146948 | HQ832586 | EF494488 | **MK088171** |
| KF112914 | MH559776 | MH426557 | KJ146952 | EF494486 | EF494487 | KR188513 |
| AY593780 | MH559805 | KF112899 | KJ146950 | JF749847 | EF117837 | KR188514 |
| AY593781 | MH559797 | AY593763 | KJ146946 | JF749845 | KJ933864 |  |

**Supplementary Table 1d: Dataset 4, 5 and 6 for lineage level diversity analysis of VP2 protein of FMDV serotype O.** According to OIE/FAO Reference Laboratoy (<https://www.wrlfmd.org/fmdv-genome/fmd-prototype-strains#panel-1412>), ME-SA topotype of serotype O is comprised of three lineages- Ind2001, PanAsia-I, PanAsia-II. 78 VP2 sequences of these three lineages were compiled in dataset 4 to analyze the lineage level diversity of O/ME-SA topotype. Topotype SEA of serotype O contains two lineages which are CAM-94 and MYA-98. Dataset 5 was generated with 5 sequences of these two lineages. O/Euro-SA topotype has three defined lineages (O1, O2, O3). Dataset 6 was prepared with VP2 sequences of three O1 and one O2 isolates, No VP2 sequence of O3 lineage was available in databank, thus this lineage was omitted in this study. Here, only the accession no. of the sequences are listed. (Bangladeshi sequences are marked as bold).

| **Dataset 4** | | | | | **Dataset 5** | **Dataset 6** |
| --- | --- | --- | --- | --- | --- | --- |
| AJ539139 | MG983697 | KJ825808 | MG983737 | MG983687 | KJ831666 | AY593815 |
| AY593824 | MG983696 | KJ825801 | MG983716 | MG983705 | KJ831680 | AY593817 |
| AH012985 | KJ825802 | KJ825809 | MG983711 | MG983706 | HQ632769 | AY593819 |
| AJ539136 | MG983721 | KJ825804 | MG983731 | MG983692 | KR401154 | M55287 |
| AJ539138 | MG983722 | KJ825805 | MG983693 | MG983688 | KR265077 |  |
| AF506822 | KJ206910 | KJ825807 | MG983740 | **KF985189** |  |  |
| HQ632770 | MG983725 | KJ825806 | KJ206908 | **KX712091** |  |  |
| JX171679 | MG983726 | MG983714 | MG983708 | MG983718 |  |  |
| MG983683 | MG983723 | MG983715 | MG983709 | MG983703 |  |  |
| MG983735 | MG983724 | MG983732 | MG983712 | MG983698 |  |  |
| MG983694 | KU291242 | MG983733 | MG983713 | MG983700 |  |  |
| KJ206909 | MG983685 | MG983734 | MG983717 | MG983701 |  |  |
| MG983695 | KJ825803 | MG983736 | MG983684 | MG983702 |  |  |
| MG983699 | MG983729 | MG983727 | MG983728 | MG983691 |  |  |
| MG983738 | MG983739 | MG983720 | MG983730 | MG983741 |  |  |
| MG983704 | MG983686 | MG983690 |  |  |  |  |

**Supplementary Table 1e: Dataset 7, 8 and 9 for lineage level diversity analysis of VP2 protein of FMDV serotype A.** Dataset 7, 8, 9 contain 35, 7 and 19 VP2 sequences of lineages under A/ASIA, A/AFRICA and A/Euro-SA topotypes, respectively. According to OIE/FAO Reference Laboratoy, ASIA topotype of serotype A is comprised of nine lineages- A22, Iran-87, Iran-96, Iran-99, Iran-05, A15, Thai-87, Sea-97, G-VII. Among them, VP2 sequences of A22, A15, Iran-87, Iran-96, Iran-05, Thai-87, Sea-97 and G-VII were available in GenBank. Dataset 7 was prepared with 35 VP2 sequences of these eight lineages to analyze the lineage level diversity of A/ASIA topotype. Topotype AFRICA of serotype A contains seven lineages which are G-I to G-VII. Seven VP2 sequences of these seven lineages were compiled in dataset 8. Dataset 9 was generated with 19 VP2 sequences of four lineages of A/Euro-SA topotype which are A5, A12, A24, A81. Here, only the accession no. of the sequences are listed.

| **Dataset 7** | | | | **Dataset 8** | **Dataset 9** | |
| --- | --- | --- | --- | --- | --- | --- |
| AY593763 | KU612816 | KF112907 | KF112919 | KF112914 | MH559779 | MH559787 |
| AY593762 | KU612822 | JF749845 | HQ268510 | KF112910 | MH746921 | MH559803 |
| AY593764 | KJ146946 | JF749844 | KF152935 | MH053305 | MH559784 | AY593768 |
| AY593765 | KU612817 | EF494488 | KR188514 | KF112916 | MH559789 | KF112899 |
| HM854025 | HQ832587 | EF494487 | KF112922 | MH053306 | MH559795 | AY593780 |
| KJ146948 | EF494486 | EF117837 | HQ832586 | AY593761 | MH559797 | AY593781 |
| KJ146952 | JF749847 | KR188513 | KY825717 | KF112925 | MH559805 | M10975 |
| HM854021 | JF749841 | KJ933864 | AY593755 |  | MH426557 | AY593752 |
| KJ146950 | KF112923 | HQ268508 |  |  | MH426574 | KF112926 |
|  |  |  |  |  | MH559776 |  |

**Supplementary Table 1f: Dataset 10 for lineage level diversity analysis of VP2 protein of FMDV serotype Asia1.** The only one topotype of serotype Asia1 which is topotype ASIA, is subdivided into 8 lineages (G-I to G-VIII). Interestingly, a new lineage (G-IX) of FMDV has been emerged in Bangladesh ([Ali et al., 2019](#_ENREF_2)) whereas previously reported sequences of Bangladesh clustered with lineage G-VIII. 42 VP2 sequences of these 9 lineages were compiled to create the dataset 10. Here, only the accession no. of the sequences are listed. (Bangladeshi sequences are marked as bold).

| **Dataset 10** | | | | | | |
| --- | --- | --- | --- | --- | --- | --- |
| **MF782478** | KT153336 | HQ268516 | AY593800 | DQ989306 | DQ989321 | DQ989307 |
| KT153338 | KT153334 | JF749849 | AY593798 | DQ989303 | DQ989318 | DQ989315 |
| KT153321 | KT153327 | HQ268513 | AY390432 | AY304994 | DQ989322 | JN006721 |
| KT153323 | KT153322 | HQ268514 | AY593797 | DQ989323 | DQ989319 | JN006720 |
| KT153364 | KT153325 | HQ268512 | AY593796 | DQ989317 | DQ989320 | JN006719 |
| KT153339 | AY593795 | HQ113233 | DQ989304 | DQ989314 | AY687334 | **MN722609** |

**Supplementary Table 1g: Dataset 11, 12 and 13 for comparative sublineage level diversity analysis of VP2 and VP1 protein of FMDV.** According to OIE/FAO Reference Laboratory Network for Foot-and-Mouth Disease, Ind2001 and PanAsia II lineage of O/ME-SA topotype of FMDV has several sublineages. Also, IRN-05 lineage of A/ASIA topotype has been reported to possess several sublineages. No other lineages were reported to be divided into sublineages according to OIE/FAO. So in our study, we analyzed the sublineage level divergence of VP2 and VP1 protein in case of Ind2001, PanAsia II and IRN-05 lineages. The most prevalent lineage circulating in Bangladesh is Ind2001 lineage of Middle East South Asia (ME-SA) topotype under serotype O. Until 2018, Ind20001 lineage of O/ME-SA topotype was subdivided into four sublineages- Ind2001a, Ind2001b, Ind2001c and Ind2001d. Recently, emergence of two new sublineages (Ind2001BD1 and Ind2001BD2) is reported by Siddique *et. al.* based on VP1 diversity analysis ([Siddique et al., 2018](#_ENREF_8)). Among the sublineages of Ind20001 lineage, Ind2001d is more common in Bangladesh and Indian subcontinent. The three others (Ind2001a, Ind2001b and Ind2001c) are exclusively found in and around Saudi Arabia. In this study, this three sublineage have been excluded for analysis feasibility and thus Ind2001d, Ind200BD1 and Ind2001BD2 were taken into account to create dataset 11 for sublineage level diversity analysis. A total of 99isolates of these three sublineages were selected and their VP2 and VP1 sequences were compiled in dataset 11 (except 8 unavailable VP1 sequences underlined in the following table). VP2 sequences of 20 isolates sequenced in the current study were included in this dataset and marked as red. . Dataset 12 was prepared with 16 FMDV polyprotein sequences of 3 sublineages under O/PanAsia II lineage. No VP2 sequences were available for the sublineage O/ME-SA/PanAsia-II^TER-08^, O/ME-SA/PanAsia-II^BAL-09^, O/ME-SA/PanAsia-II^QOM-15^, O/ME-SA/PanAsia-II^SAN-09^, O/ME-SA/PanAsia-II^KAT-15^. Thus these sublineages were excluded and the dataset was generated with O/ME-SA/PanAsia-II^ANT-10^, O/ME-SA/PanAsia-II^FAR-09^ and O/ME-SA/PanAsia-II^PUN-10^ sublineages. IRN-05 lineage of A/ASIA topotype carries 11 sublieages (A/IRN-05^AFG-07^, A/IRN-05^ARD-07^, A/IRN-05^EZM-07^, A/IRN-05^BAR-08^ , A/IRN-05^FAR-09^, A/IRN-05^KSS-09^, A/IRN-05^ESF-10^, A/IRN-05^HER-10^, A/IRN-05^SIS-10^, A/IRN-05^QAZ-11^ and A/IRN-05^SIS-12^). But VP2 sequences were available for only A/IRN-05^AFG-07^, A/IRN-05^ARD-07^ and A/IRN-05^BAR-08^ sublineages. Thus dataset 13 was created with 5 polyprotein sequences of these three sublineages. Here, only the accession no. of the sequences are listed. (Bangladeshi sequences are marked as bold).

| **Dataset 11** | | | | | **Dataset 12** | **Dataset 13** |
| --- | --- | --- | --- | --- | --- | --- |
| **MN722610, KY077610** | **MN722616,**  **KY077620** | **MN722613,** **KY077617** | **MN722626** | **MN722625** | KR149704 | JF721438 |
| **MN722614,** **KY077618** | **MN722617,** **KY077621** | **MN722619,** **KY077623** | **MN722627** | **MN722628** | JX170756 | JF721436 |
| **MN722615,** **KY077619** | **MN722618,** **KY077622** | **MN722620,** **KY077627** | **MN722629** | **MN722630** | KM268895 | JF721439 |
| **MN722611, KY077613** | **MN722612,** **KY077615** | **MN722622,** **KY077602** | **MN722631** | **MN722632** | KJ831689 | JN006722 |
| **MN722621,** **KJ175183** | **MN722623,**  **KY077604** | **MN722624,** **KY077605** | **MK071699** | **KF985189** | KR149715 | KF112924 |
| **KX712091** | **MG983684** | **KY077611** | MG983685 | MG983706 | KJ831686 |  |
| MG983683 | KJ825802 | MG983710 | MG983686 | KJ825804 | KJ831688 |  |
| KJ825801 | MG983704 | MG983718 | MG983687 | KJ825805 | KJ831726 |  |
| KU291242 | MG983705 | MG983719 | MG983688 | KJ825806 | KU365846 |  |
| KJ206910 | MG983740 | MG983720 | KJ206908 | KJ825807 | KY077605 |  |
| KJ825809 | MG983702 | MG983727 | MG983690 | MG983692 | KU365845 |  |
| MG983721 | MG983691 | MG983728 | KJ825803 | MG983693 | JX170745 |  |
| MG983707 | MG983689 | MG983729 | MG983699 | MG983694 | KR149717 |  |
| MG983697 | MG983698 | MG983730 | MG983700 | KJ206909 | KJ831727 |  |
| KJ825808 | MG983741 | MG983738 | MG983701 | MG983695 | KR149717 |  |
| MG983739 | MG983703 | MG983722 | MG983723 | MG983696 | KR149718 |  |
| MG983708 | MG983713 | MG983724 | MG983732 | MG983717 |  |  |
| MG983709 | MG983714 | MG983725 | MG983733 | MG983736 |  |  |
| MG983711 | MG983715 | MG983726 | MG983734 | MG983737 |  |  |
| MG983712 | MG983716 | MG983731 | MG983735 |  |  |  |

**Supplementary Table 1h: Dataset 14 for identifying the conserved fragments in VP2 protein of FMDV.** A total of 360 FMDV VP2 sequences representing the isolates of Asian territory were taken into account. Representative data from all FMDV subgroups that were available in databank were included. No VP2 sequences of the following subgroups were available in databank- O/ISA-1, O/ISA-2, O/ME-SA/PanAsia-II^TER-08^, O/ME-SA/PanAsia-II^BAL-09^, O/ME-SA/PanAsia-II^QOM-15^, O/ME-SA/PanAsia-II^SAN-09^, O/ME-SA/PanAsia-II^KAT-15^, A/IRN-99, A/IRN-05^EZM-07^, A/IRN-05^FAR-09^, A/IRN-05^KSS-09^, A/IRN-05^ESF-10^, A/IRN-05^HER-10^, A/IRN-05^SIS-10^, A/IRN-05^QAZ-11^, A/IRN-05^SIS-12^ . Due to unavailability of VP2 sequences, this subgroups were excluded in this study. All the 49 Asian countries were taken into account and representative data from all countries were included except few from where no VP2 sequences were reported. Among the total 360 VP2 sequences, 137 belong to serotype O, 118 belong to serotype A and 105 belong to serotype Asia1. Here, only the accession no. of the sequences are listed. (Bangladeshi sequences are marked as bold).

| **Dataset 14** | | | | | | |
| --- | --- | --- | --- | --- | --- | --- |
| **KF985189** | MG983741 | KY322671 | HQ832590 | KU612850 | KT153337 | HQ268516 |
| **MN722621** | **KY077611** | KR401193 | FJ617243 | KU612857 | KT153339 | JN247565 |
| **MN722623** | MG983727 | KJ831695 | KU612842 | KJ146937 | KT153335 | JQ818553 |
| KJ825809 | MG983728 | KF112882 | KJ146954 | KJ146948 | KT153338 | AY687334 |
| KJ825804 | MG983729 | KY825723 | FJ617246 | KJ146950 | KT153336 | DQ989307 |
| KJ825805 | MG983719 | KJ831696 | KU612846 | KJ146946 | KT153334 | DQ989305 |
| KJ825807 | MG983703 | KM243131 | HQ832591 | KJ146952 | KT153366 | DQ989312 |
| MG983715 | MG983691 | KR401181 | KU612845 | KU612817 | KT153364 | DQ989316 |
| MG983736 | MG983738 | KR401154 | FJ617244 | KU612816 | KT153360 | DQ989315 |
| MG983737 | MG983739 | KR265077 | KU612840 | KU612822 | KT153353 | DQ989319 |
| MG983716 | MG983689 | KR401195 | KU612862 | HM854021 | MF372125 | DQ989321 |
| MG983711 | MG983707 | KY412560 | KU612863 | HQ832587 | KT153342 | DQ989317 |
| KJ825806 | MG983710 | LC149719 | KU612865 | KU612837 | KT153363 | DQ989314 |
| MG983693 | MG983717 | LC149717 | FJ617242 | HQ832586 | KT153359 | DQ989318 |
| MG983740 | **MK071699** | LC149718 | KU612844 | KU612884 | KT153358 | DQ989322 |
| KJ825808 | AJ539139 | LC149716 | KU612833 | KU612883 | KT153361 | DQ989320 |
| KJ825801 | AY593824 | KR265082 | KU612829 | KF112907 | KT153367 | DQ989323 |
| KJ825803 | AJ539136 | KJ831666 | HQ832578 | JF749841 | KT153354 | HQ268515 |
| MG983686 | AJ539138 | KJ831680 | KU612826 | KF112923 | KT153349 | MG372731 |
| MG983690 | AF506822 | HQ632769 | KU612832 | JF749847 | KT153347 | AY593800 |
| MG983732 | AH012985 | KJ831682 | HQ832579 | EF494486 | KT153348 | AY593798 |
| MG983734 | HQ268525 | KR149713 | KU612824 | JF749845 | KT153346 | JF739177 |
| MG983733 | HQ632770 | AY593813 | KU612827 | JF749844 | KT153362 | MG372731 |
| MG983731 | JX171679 | AF308157 | KU612828 | KF112922 | KT153357 | MF063056 |
| MG983714 | KP720595 | HQ268518 | HQ832580 | EF494488 | KT153355 | MF063055 |
| **MN722624** | KJ606980 | KJ415245 | FJ617245 | EF494487 | KT153343 | MF063054 |
| KJ825802 | FJ175665 | AY317098 | HQ832582 | JF721438 | KT153365 | AY593797 |
| MG983725 | FJ175666 | EU400597 | KU612831 | JF721436 | KT153350 | AY593796 |
| MG983721 | KJ831694 | KJ831677 | KU612834 | JF721439 | KT153368 | KP822943 |
| KJ206910 | KR149718 | KJ415243 | FJ617251 | JN006722 | KT153351 | DQ989304 |
| MG983722 | KM268895 | HQ268521 | HQ832583 | KF152935 | KT153326 | DQ989303 |
| MG983726 | KR149704 | EF175732 | KU612835 | AY593755 | KT153325 | AY304994 |
| MG983723 | KU365845 | **KJ754939** | HQ832581 | AY593763 | KT153341 | DQ989306 |
| MG983724 | JX170756 | KU612879 | FJ617247 | AY593764 | JN247566 | AY390432 |
| KJ206908 | KJ831726 | KU612876 | KU612866 | AY593762 | KT153327 | KC412634 |
| MG983712 | JX170757 | KU612872 | FJ617248 | KY825717 | KT153332 | FJ906802 |
| MG983713 | KR149715 | KU612877 | KU737528 | AY593765 | KT153345 | HQ631363 |
| MG983708 | KU365846 | KU612875 | FJ617250 | HM854022 | JN247567 | EF614458 |
| MG983709 | JX170745 | KU612868 | KJ146920 | KJ933864 | DQ989310 | GU125645 |
| MG983685 | KJ606981 | KU612867 | KU612856 | HQ268508 | DQ989311 | DQ989313 |
| **MG983684** | KJ831686 | KU612878 | KU612858 | HQ268510 | EF134952 | AY593795 |
| MG983687 | KJ831727 | KU612881 | KM189190 | KF112919 | MF372126 | AJ251478 |
| MG983705 | KJ831693 | KU612880 | KM189184 | AY593755 | DQ989309 | HQ632774 |
| MG983688 | AY593828 | KU612869 | KM189183 | KR188513 | DQ989308 | GU125646 |
| MG983706 | AY593812 | KU612874 | HQ666878 | KR188514 | EF149010 | JN006719 |
| MG983692 | AY593811 | KU612882 | HQ666887 | KR188515 | JF749849 | JN006721 |
| MG983704 | KR149707 | KU612871 | KU612852 | **MN722609** | KY825721 | JN006720 |
| **KX712091** | KR149706 | **MK088171** | KU612851 | **MF782478** | HQ268512 | KM268898 |
| MG983718 | KR139753 | KU127247 | HQ666886 | KT153322 | HQ268514 |  |
| LC320038 | KP822947 | KU612848 | KU612853 | KT153324 | HQ268513 |  |
| MG983720 | KJ831692 | KU612847 | KU612855 | KT153321 | HQ113233 |  |
| MG983730 | KR265083 | HQ832592 | HQ666879 | KT153323 | AY687333 |  |

**Supplementary Table 2: Previously reported antigenic sites of VP2.**

| **Antigenic Sites** | **References** |
| --- | --- |
| **N-terminal (1-14)** | Freiberg *et al.* showed that epitopes for the monoclonal antibodies raised against FMDV isolates recommended for type-independent antigen detection by ELISA reside at the N-terminus of FMDV capsid protein VP2 ([Freiberg et al., 2001](#_ENREF_7)). Monoclonal antibody developed by Yang *et al.* also showed reactivity with N-terminal of VP2 suggesting the region to be an immunodominant site ([Yang et al., 2007](#_ENREF_10)). |
| **22-36 motif** | 22-36 motif of FMDV is reported to play critical role in forming polyprotein cleavage pocket and virion infectivity by interaction with host cell ([Carrillo et al., 2005](#_ENREF_5)) suggesting it’s possibility to retain potential antigenic sites. |
| **B-C (70-80)** | VP2 comprises neutralizing antigenic site II of Foot and mouth disease virus which resides in the B-C loop of VP2 protein. Crystallographic data and studies utilizing neutralization resistant variants have shown that amino acids 70-80 of the VP2 B-C loop are located in close proximity to the VP1 G-H loop ([Acharya et al., 1989](#_ENREF_1)). |
| **E-F loops (132-135)** | Another important loop in VP2 is the E-F loop which is consisted of amino acid residues 132-135 ([Bai et al., 2010](#_ENREF_4)), also forms an antigenic site ([Freiberg et al., 2001](#_ENREF_7)) . |
| **T-cell epitopes** | Three T-cell epitopes was identified at stretches 48-68, 114-132, 179-187 of VP2 by Filgueira *et al.* using murine model ([Filgueira et al., 2000](#_ENREF_6)) ([Carrillo et al., 2005](#_ENREF_5)). The epitopes are mentioned as T cell epitope I (48-68), T cell epitope II (114-131) and T cell epitope III (179-187) in this study. As amino acid 132 constitutes both part of E-F loop and T cell epitope II, this has been discussed under E-F loop here. |
| **Antigenically Critical amino acids (100, 172, 188 and 191)** | Four amino acids in VP2 (56, 100, 131 and 172) were found to be under positive selection by Subramaniam *et al.* which suggests their importance in the antigenicity of FMDV ([Subramaniam et al., 2015](#_ENREF_9))**.** Asfor *et. al.,* reported that VP2-72, 74, 78, 188 and 191 in the three fold axis of the virion structure of FMDV are antigenic ([Asfor et al., 2014](#_ENREF_3)). Among these reported sites, amino acids 72, 74 and 78 resides in the B-C loop, 56 and 131 in T cell epitope I and II respectively. The rest four other amino acids (100, 172, 188 and 191) are part of antigenically crucial discontinuous epitopes. In the present study, these sites are mentioned as critical amino acids I, II, III and IV as major mutation in this sites can affect the antigenicity of the virus. |

**Supplementary Table 3: Mutations in the antigenic regions of VP2 protein of three FMDV serotypes observed by analysis of Dataset 1 (Supplementary Table 1a).** For each position in VP2, the most common amino acid is placed at the top and the mutated different amino acids are shown down to the most common one. Only the previously reported antigenic sites of VP2 were taken into account for mutation analysis.

| **Antigenic sites** | **Position** | **Serotype O** | **Serotype A** | **Serotype Asia1** |
| --- | --- | --- | --- | --- |
| **N- terminal (1-14)** | **7** | **T** | **T** | **T** |
|  |  | **A , I** |  |  |
|  | **9** | **L** | **L** | **L** |
|  |  |  |  | **M, F** |
|  | **10** | **L** | **L** | **L** |
|  |  |  |  | **F** |
| **22-36 motif** | **23** | **I** | **T** | **T** |
|  |  | **T** |  |  |
| **T cell epitope I (48-68)** | **56** | **V** | **V** | **Q** |
|  |  | **L, I** | **G, I** | **H, T, I, V** |
|  | **64** | **T** | **K** | **K** |
|  |  |  |  | **N** |
|  | **65** | **H** | **H** | **H** |
|  |  | **R** | **F** |  |
| **B-C loop (70-80)** | **70** | **V** | **T** | **T** |
|  |  |  |  | **A** |
|  | **71** | **T** | **T** | **P** |
|  |  |  |  | **T, S, Q** |
|  | **72** | **S** | **D** | **N** |
|  |  |  | **E** | **D, S** |
|  | **73** | **D** | **K** | **L** |
|  |  |  |  | **K** |
|  | **74** | **P** | **A** | **A** |
|  |  | **S** | **P, S** | **P, S, E** |
|  | **77** | **R** | **H** | **H** |
|  |  |  | **Y** | **Y** |
|  | **78** | **C** | **L** | **C** |
|  |  | **Y** |  | **L** |
|  | **79** | **H** | **E** | **H** |
|  |  | **Y** | **V, K** | **Y, C, E** |
|  | **80** | **L** | **K** | **Y** |
|  |  | **V, M** |  | **K** |
| **Critical Amino Acid I** | **100** | **Y** | **Y** | **Y** |
|  |  |  |  | **T, N** |
| **T cell epitope II (114-131)** | **125** | **M** | **M** | **L** |
|  |  |  |  | **M** |
|  | **129** | **L** | **W** | **L** |
|  |  |  | **R** | **W** |
|  | **130** | **C** | **K** | **K** |
|  |  | **Y** | **T** | **E, R** |
|  | **131** | **S** | **E** | **E** |
|  |  | **P** | **D, K, H, G** | **S, A, N, K, T** |
| **E-F Loop (132-135)** | **132** | **I** | **F** | **L** |
|  |  |  |  | **F** |
|  | **133** | **Q** | **T** | **D** |
|  |  | **D, N** | **D, S** | **T** |
|  | **134** | **K** | **L** | **T** |
|  |  |  | **T, P, A** | **L, I, E** |
| **Critical Amino Acid II** | **172** | **K** | **K** | **E** |
|  |  |  |  | **A, Q, M, V, K** |
| **T cell epitope III (179-187)** | **181** | **V** | **V** | **V** |
|  |  |  |  | **A** |
|  | **184** | **V** | **V** | **V** |
|  |  |  | **I** | **L** |
|  | **185** | **A** | **S** | **A** |
|  |  | **T** |  | **S** |
| **Critical Amino Acid III** | **188** | **T** | **T** | **T** |
| **Critical Amino Acid IV** | **191** | **T** | **S** | **T** |
|  |  | **N** | **N, T ,G** | **S** |

**References**

Acharya, R., E. Fry, D. Stuart, G. Fox, D. Rowlands and F. Brown (1989). "The three-dimensional structure of foot-and-mouth disease virus at 2.9 Å resolution." Nature **337**(6209): 709.

Ali, M. R., A. Alam, M. Al Amin, M. A. Siddique, M. Sultana and M. A. Hossain (2019). "Emergence of Novel Lineage of Foot-and-Mouth Disease Virus Serotype Asia1 BD-18 (G-IX) in Bangladesh." bioRxiv: 604892.

Asfor, A. S., S. Upadhyaya, N. J. Knowles, D. P. King, D. J. Paton and M. Mahapatra (2014). "Novel antibody binding determinants on the capsid surface of serotype O foot-and-mouth disease virus." The Journal of general virology **95**(Pt 5): 1104.

Bai, X., H. Bao, P. Li, P. Sun, W. Kuang, Y. Cao, Z. Lu, Z. Liu and X. Liu (2010). "Genetic characterization of the cell-adapted PanAsia strain of foot-and-mouth disease virus O/Fujian/CHA/5/99 isolated from swine." Virology journal **7**(1): 208.

Carrillo, C., E. Tulman, G. Delhon, Z. Lu, A. Carreno, A. Vagnozzi, G. Kutish and D. Rock (2005). "Comparative genomics of foot-and-mouth disease virus." Journal of virology **79**(10): 6487-6504.

Filgueira, M. P., A. Wigdorovitz, A. Romera, P. Zamorano, M. Borca and A. Sadir (2000). "Detection and characterization of functional T-cell epitopes on the structural proteins VP2, VP3, and VP4 of foot and mouth disease virus O1 Campos." Virology **271**(2): 234-239.

Freiberg, B., B. Höhlich, B. Haas, A. Saalmüller, E. Pfaff and O. Marquardt (2001). "Type-independent detection of foot-and-mouth disease virus by monoclonal antibodies that bind to amino-terminal residues of capsid protein VP2." Journal of virological methods **92**(2): 199-205.

Siddique, M., M. Ali, A. Alam, H. Ullah, A. Rahman, R. Chakrabarty, M. Amin, S. Hoque, S. Nandi and M. Sultana (2018). "Emergence of two novel sublineages Ind2001 BD 1 and Ind2001 BD 2 of foot‐and‐mouth disease virus serotype O in Bangladesh." Transboundary and emerging diseases **65**(4): 1009-1023.

Subramaniam, S., J. K. Mohapatra, B. Das, G. K. Sharma, J. K. Biswal, S. Mahajan, J. Misri, B. B. Dash and B. Pattnaik (2015). "Capsid coding region diversity of re-emerging lineage C foot-and-mouth disease virus serotype Asia1 from India." Archives of virology **160**(7): 1751-1759.

Yang, M., A. Clavijo, R. Suarez-Banmann and R. Avalo (2007). "Production and characterization of two serotype independent monoclonal antibodies against foot-and-mouth disease virus." Veterinary immunology and immunopathology **115**(1-2): 126-134.
