# Supplementary material for: In silico Evolutionary Divergence Analysis Suggests the Potentiality of Capsid Protein VP2 in Serotype-Independent Foot-and-Mouth Disease Virus Detection
Source: Front Vet Sci. 2020 Sep 25;7:592. doi: 10.3389/fvets.2020.00592 (PMC7546019; doi:10.3389/fvets.2020.00592)
Supplement: Supplementary file 2 [file Data_Sheet_2.docx]

**Supplementary Figures**

**
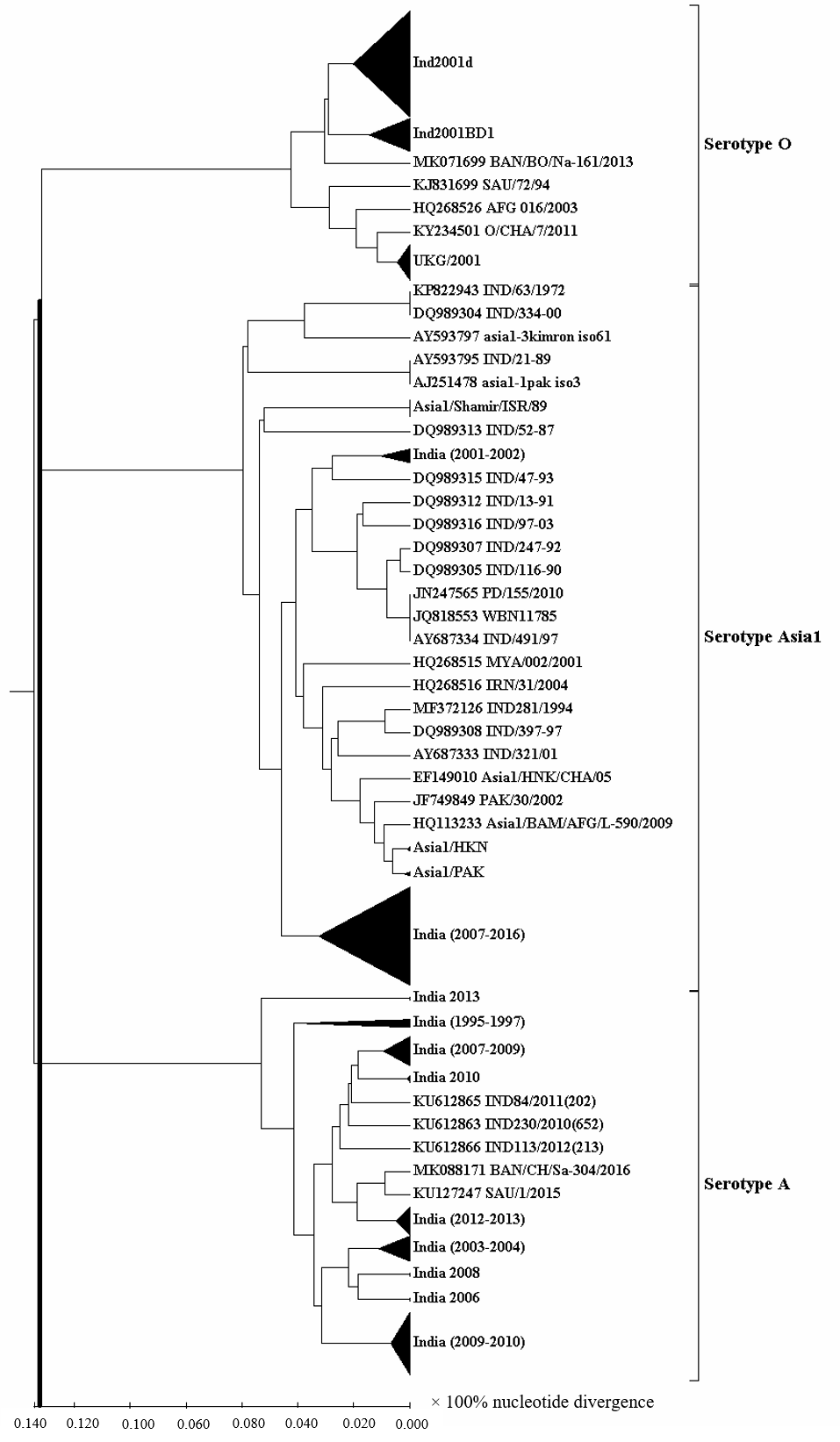
**

**Supplementary Figure 1: VP2 based phylogenetic study for serotype level divergence analysis of FMDV.** The UPGMA tree was constructed based on 279 VP2 nucleotide sequences of serotype O, A and Asia1. The black vertical line delineates a cut-off value of (0.140×100%) VP2 nucleotide divergence between two closest serotypes (O and Asia1). Sequences within same clusters are compressed for better visualization.

**
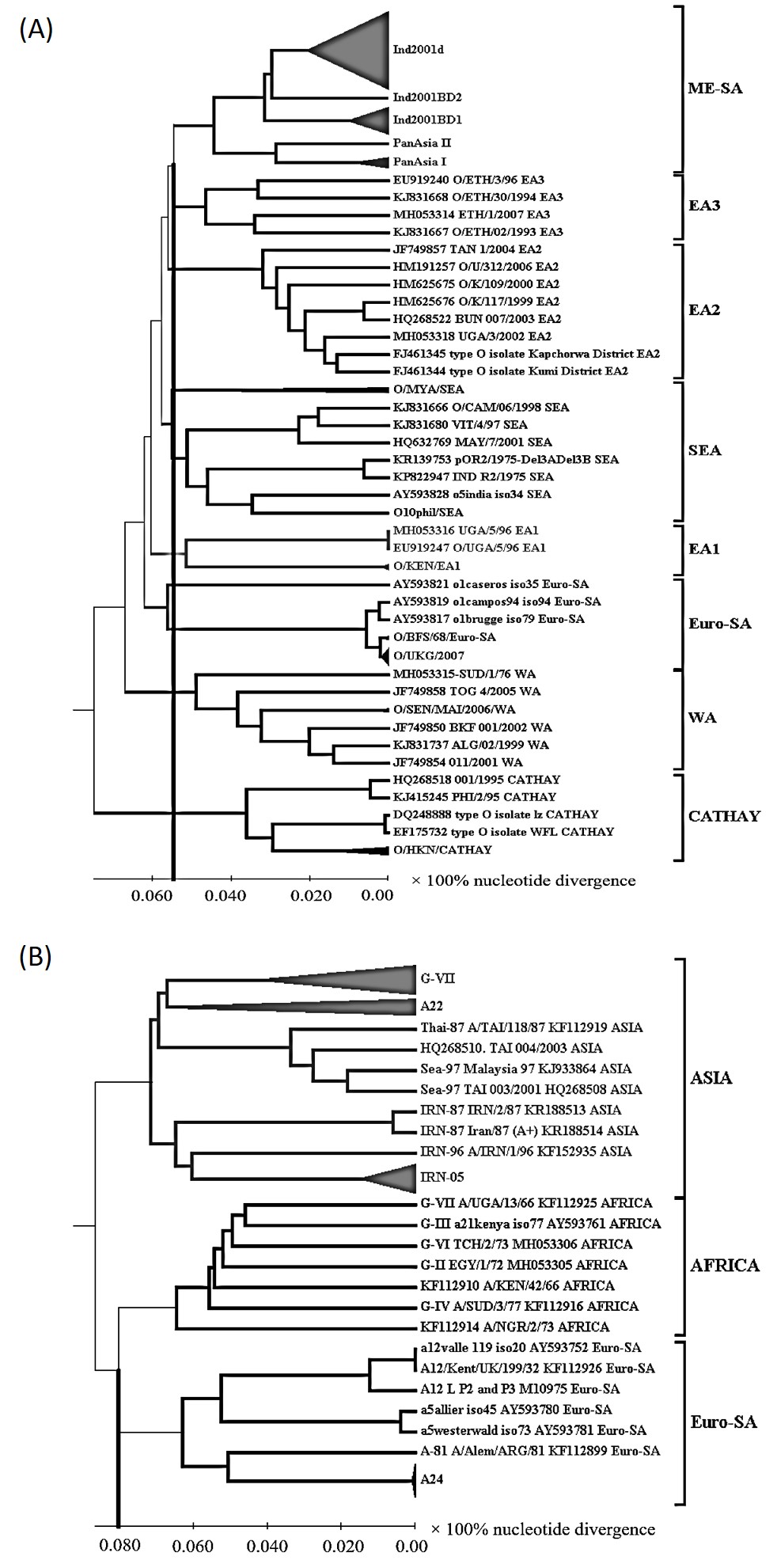
**

Supplementary Figure 2: VP2 based phylogenetic study for topotype level divergence analysis of FMDV. (A) UPGMA phylogenetic tree of eight topotypes of FMDV serotype O using 140 VP2 nucleotide sequences. The black vertical line delineates a cut-off value of (0.055×100%) VP2 nucleotide divergence between two closest topotypes (ME-SA and EA-3). (B) UPGMA phylogenetic tree of three topotypes of FMDV serotype A using 62 VP2 nucleotide sequences. The black vertical line delineates a cut-off value of (0.080×100%) VP2 nucleotide divergence between two closest topotypes (AFRICA and Euro-SA). Sequences within same clusters are compressed for better visualization.

**
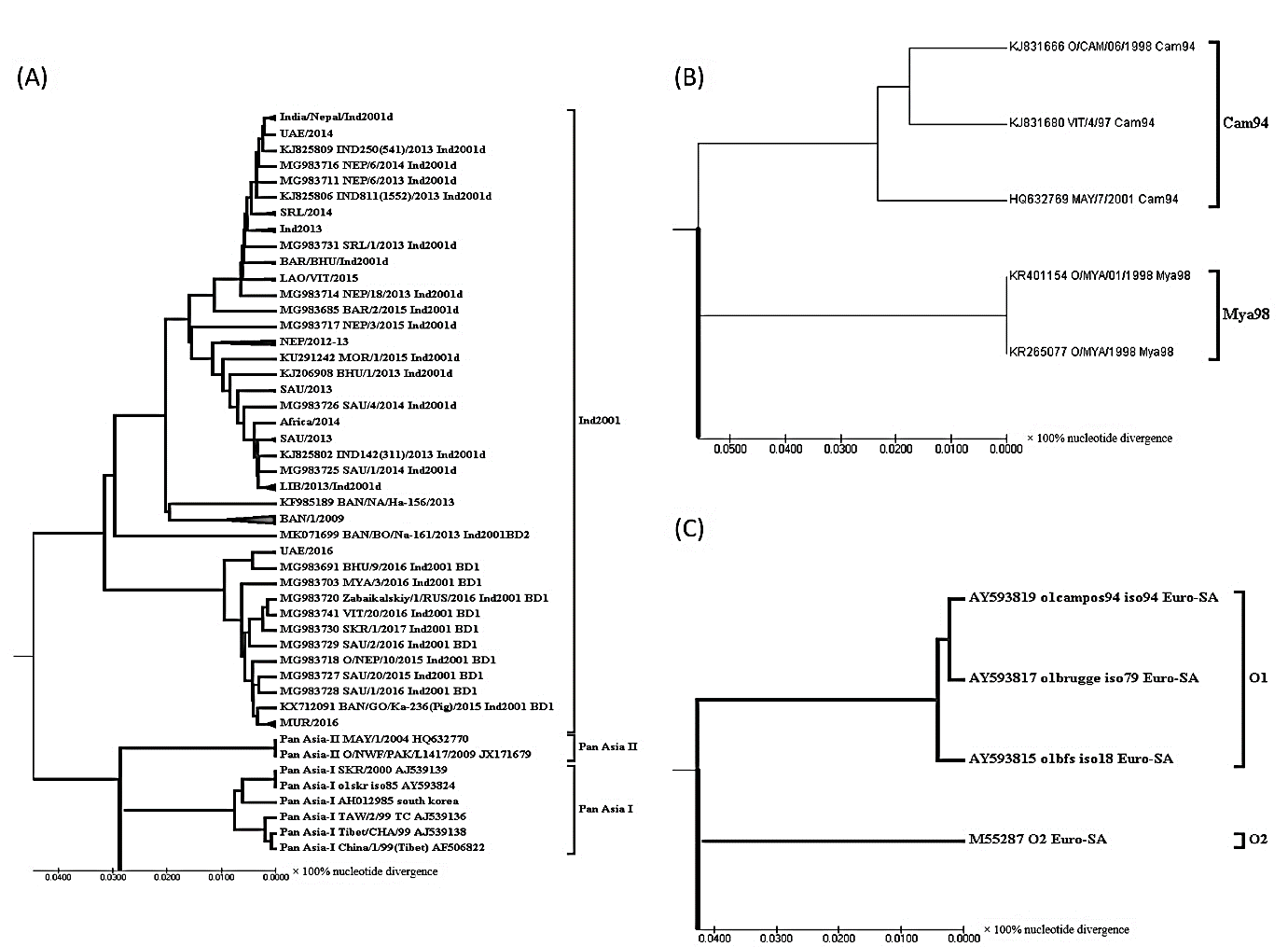
**

**Supplementary Figure 3: VP2 based phylogenetic study for lineage level divergence analysis of FMDV serotype O. (A) UPGMA phylogenetic tree of three lineages under ME-SA topotype of FMDV serotype O using 78 VP2 nucleotide sequences.** The black vertical line delineates a cut-off value of (0.029×100%) VP2 nucleotide divergence between two closest lineages (PanAsia I and PanAsia II**). (B) UPGMA phylogenetic tree of two lineages under SEA topotype of FMDV serotype O using five VP2 nucleotide sequences.** The black vertical line delineates a cut-off value of (0.055×100%) VP2 nucleotide divergence. **(C) UPGMA phylogenetic tree of two lineages under Euro-SA topotype of FMDV serotype O using four VP2 nucleotide sequences.** The black vertical line delineates a cut-off value of (0.0425×100%) VP2 nucleotide divergence. Sequences within same clusters are compressed for better visualization.

**
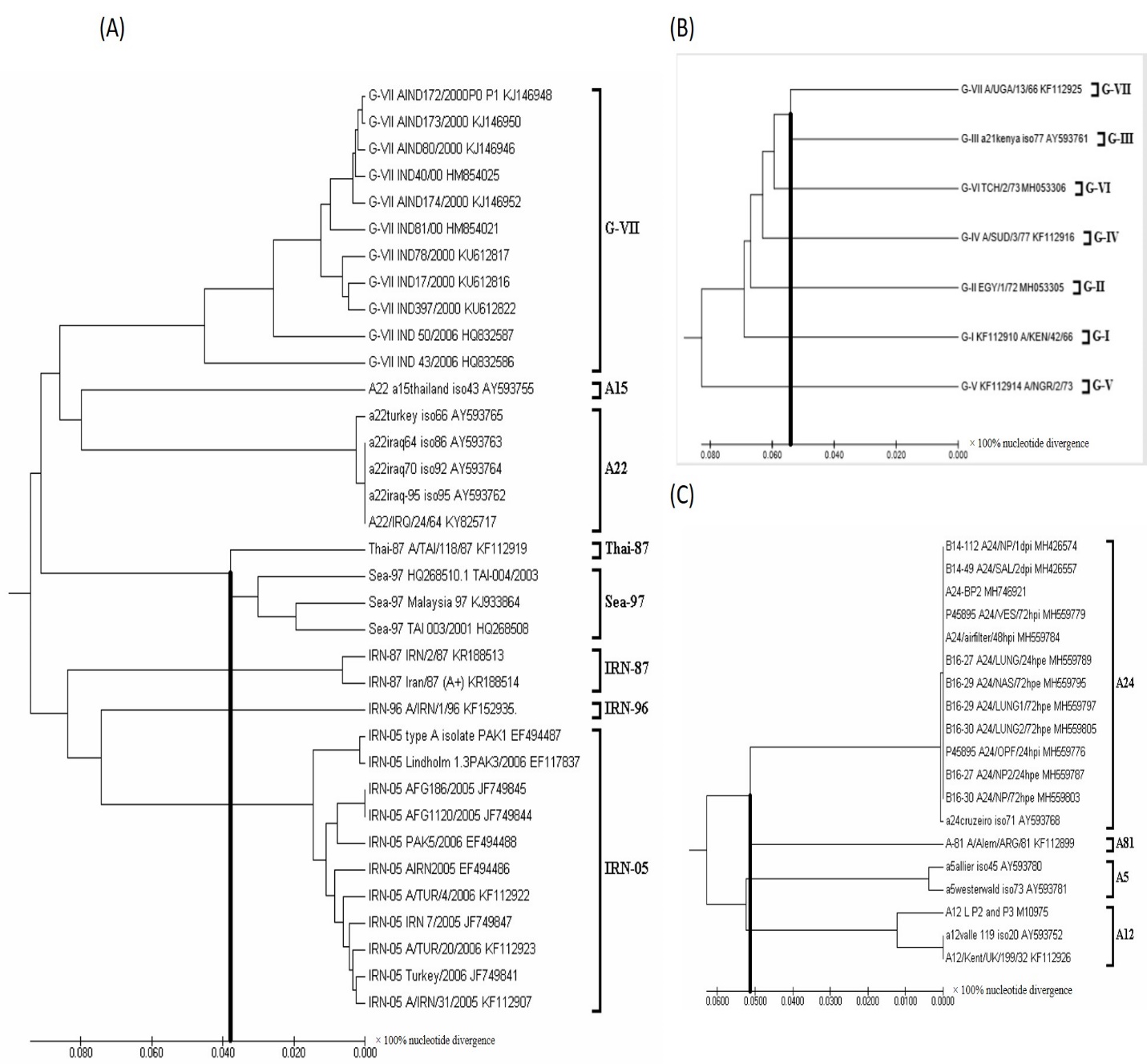
**

Supplementary Figure 4: VP2 based phylogenetic study for lineage level divergence analysis of FMDV serotype A. (A) UPGMA phylogenetic tree of eight lineages under ASIA topotype of FMDV serotype A using 35 VP2 nucleotide sequences. The black vertical line delineates a cut-off value of (0.039×100%) VP2 nucleotide divergence between two closest lineages (Thai-87 and Sea-97). (B) UPGMA phylogenetic tree of seven lineages under AFRICA topotype of FMDV serotype A using seven VP2 nucleotide sequences. The black vertical line delineates a cut-off value of (0.054×100%) VP2 nucleotide divergence between two closest lineages (G-III and G-VII). (C) UPGMA phylogenetic tree of four lineages under Euro-SA topotype of FMDV serotype A using 19 VP2 nucleotide sequences. The black vertical line delineates a cut-off value of (0.051×100%) nucleotide divergence between two closest lineages (A24 and A81).

**
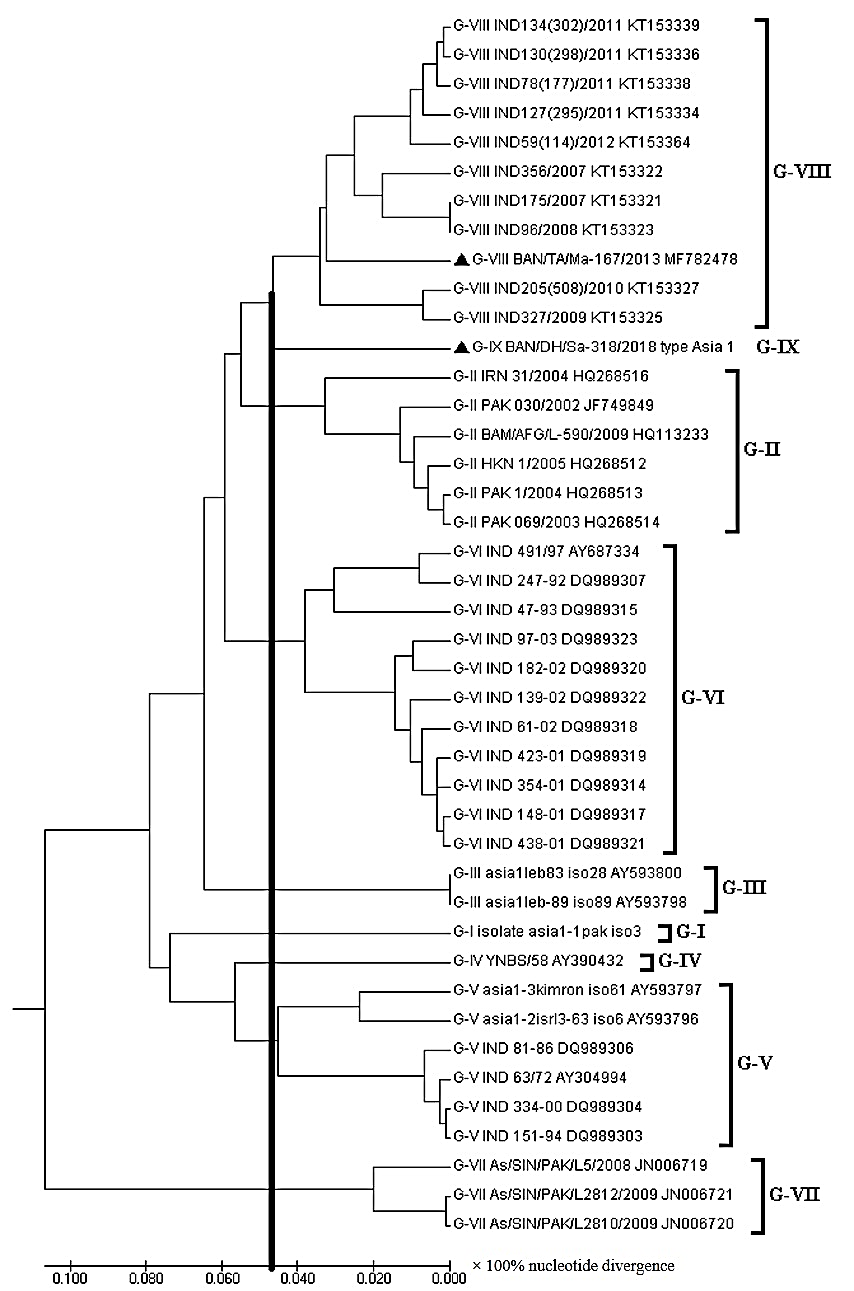
**

**Supplementary Figure 5: VP2 based phylogenetic study for lineage level divergence analysis of FMDV serotype Asia1.** The UPGMA tree was constructed based on 42 VP2 nucleotide sequences of nine lineages under ASIA topotype of FMDV serotype Asia1. The black vertical line delineates a cut-off value of (0.048×100%) VP2 nucleotide divergence between two closest lineages (G-VIII and G-IX).

**
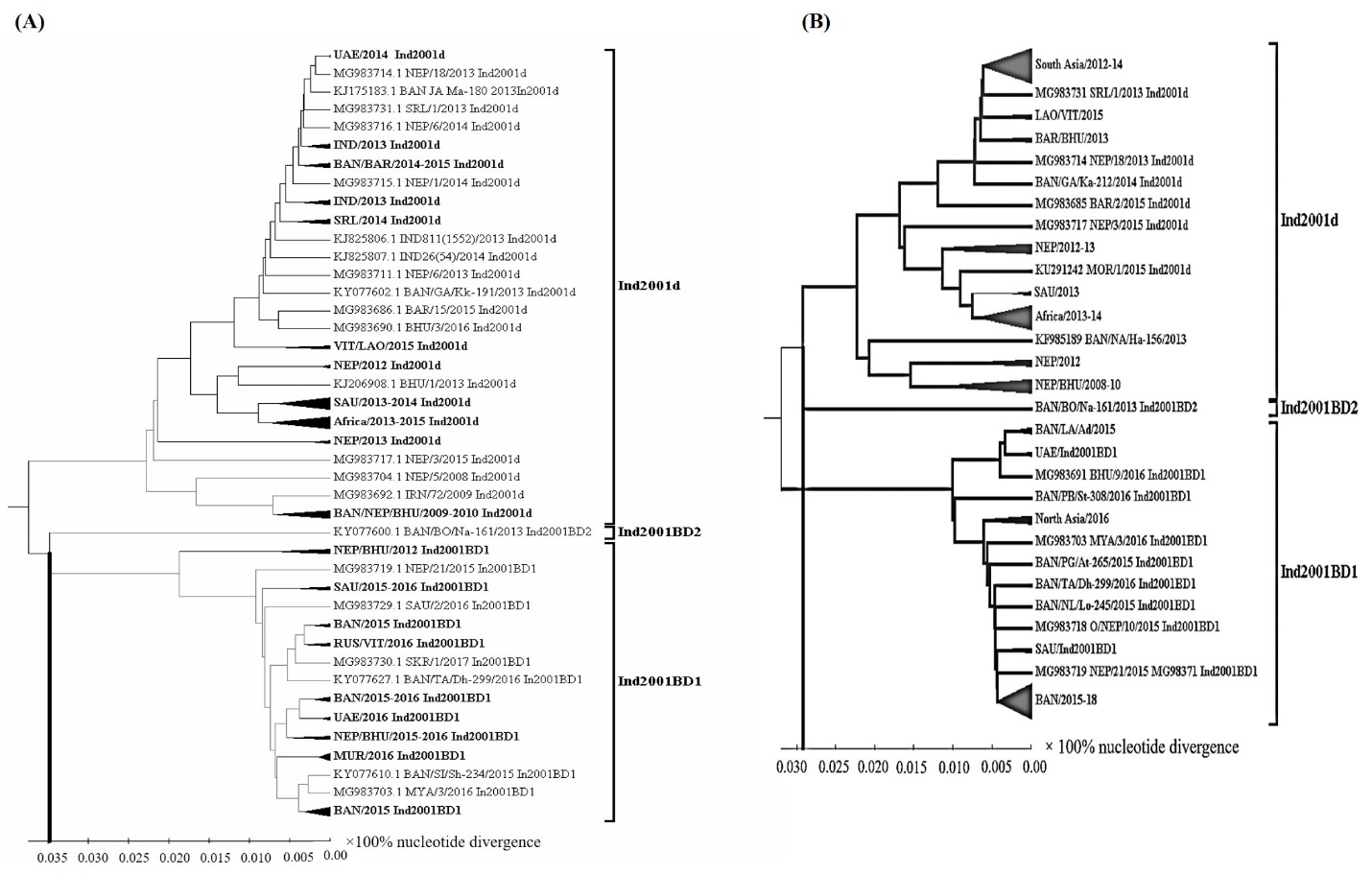
**

**Supplementary Figure 6: Comparative phylogenetic study for sublineage level divergence analysis of FMDV O/Ind2001 lineage based on VP1 (A) and VP2 (B) nucleotide sequences.** The UPGMA trees were constructed based on 99 VP2 nucleotide sequences and 91 VP1 nucleotide sequences (due to unavailability of respective 8 VP1 sequences in the databank) of three sublineages under Ind2001 lineage of O/ME-SA topotype. The black vertical lines delineate a cut-off value of (0.035×100%) for VP1 and (0.029×100%) for VP2 nucleotide divergence between two closest sublineages. Ind2001BD1 and Ind2001BD2 was found to be the closest sublineages in VP1 based phylogeny while VP2 based phylogeny showed Ind2001d and Ind2001BD2 to be the closest relative. Sequences within same clusters are compressed for better visualization.


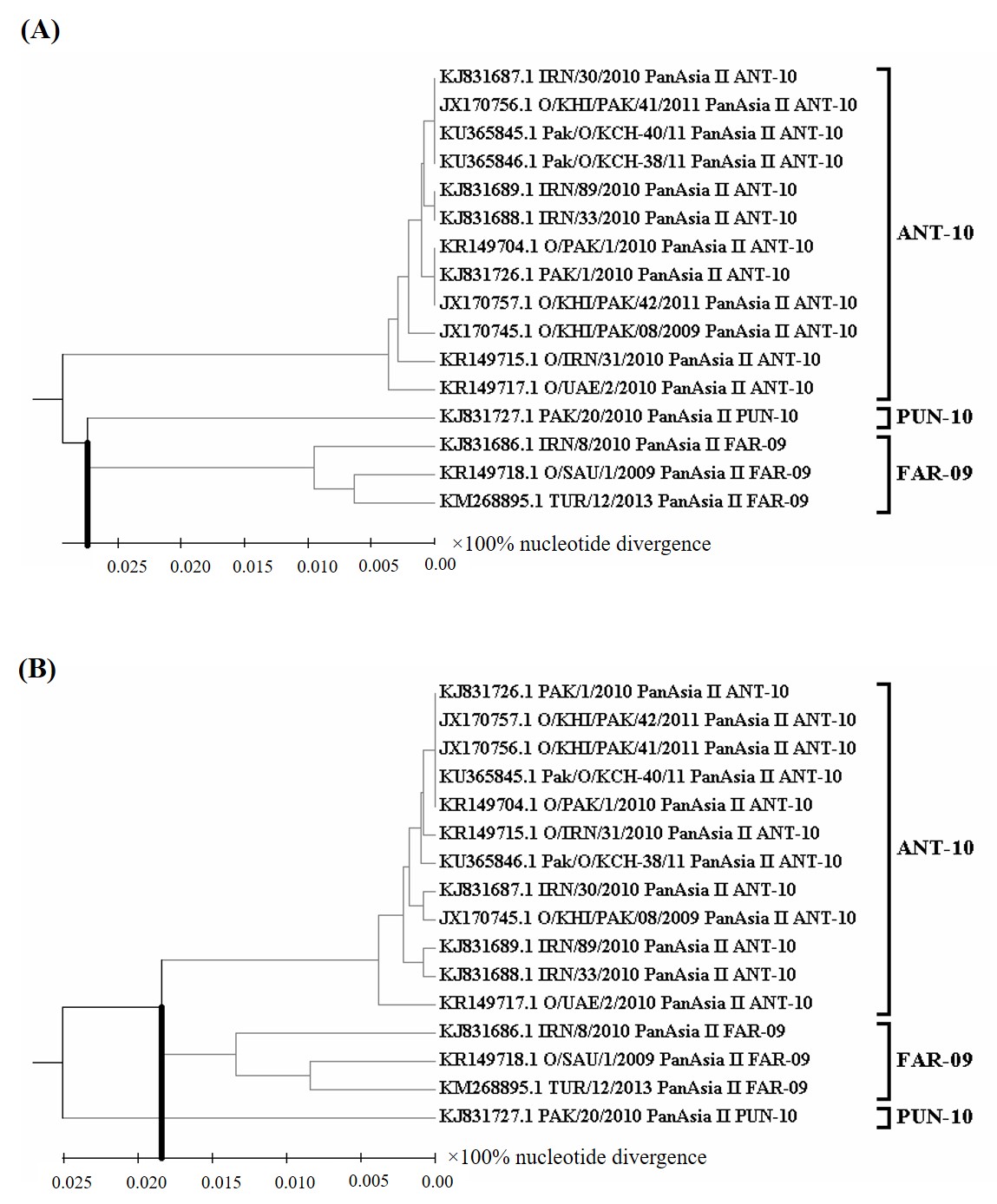


**Supplementary Figure 7: Comparative phylogenetic study for sublineage level divergence analysis of FMDV O/PanAsia II lineage based on VP1 (A) and VP2 (B) nucleotide sequences.** The UPGMA trees were constructed based on 16 VP1 and VP2 nucleotide sequences of three sublineages under PanAsia II lineage of O/ME-SA topotype. The black vertical lines delineate a cut-off value of (0.027×100%) for VP1 and (0.018×100%) for VP2 nucleotide divergence between two closest sublineages (PUN-10 and FAR-09 in case of VP1; ANT-10 and FAR-09 in case of VP2).


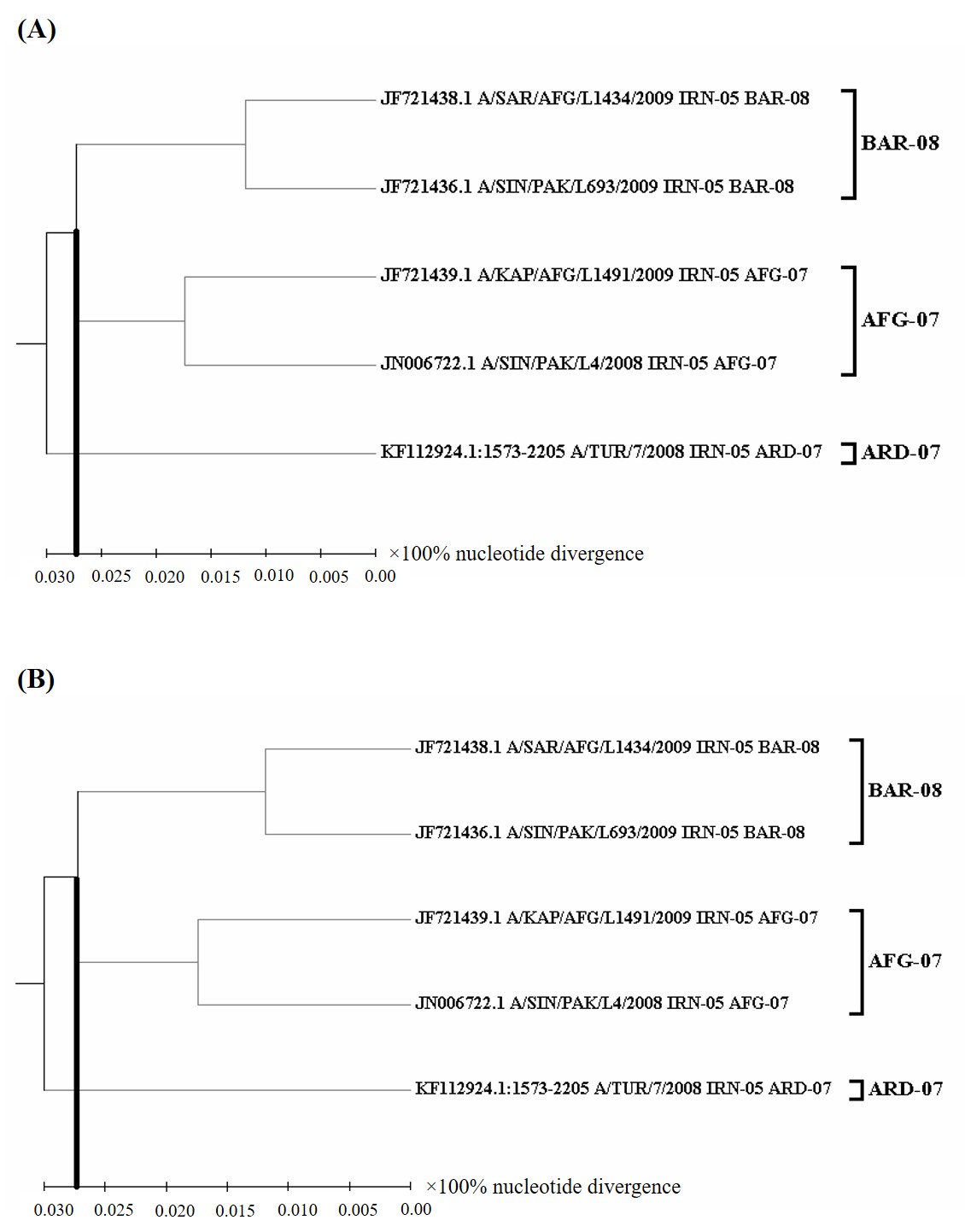


**Supplementary Figure 8: Comparative phylogenetic study for sublineage level divergence analysis of FMDV A/IRN-05 lineage based on VP1 (A) and VP2 (B) nucleotide sequences.** The UPGMA trees were constructed based on 5 VP1 and VP2 nucleotide sequences of three sublineages under IRN-05 lineage of A/ASIA topotype. The black vertical lines delineate a cut-off value of (0.027×100%) for VP1 and (0.027×100%) for VP2 nucleotide divergence between two closest sublineages (BAR-08 and AFG-07 in each cases).

**
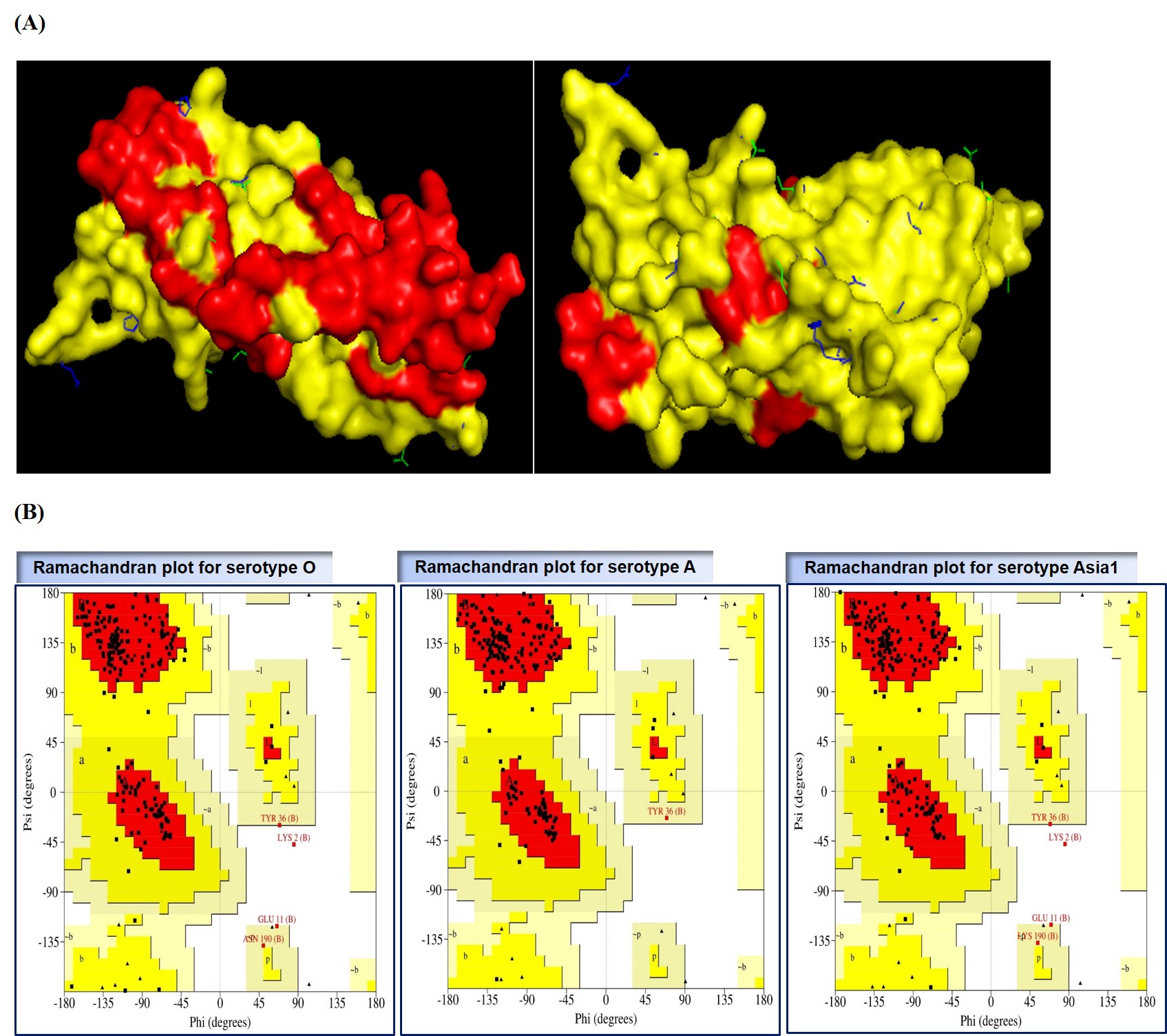
**

Supplementary Figure 9: (A) Three-dimensional structure of superimposed VP2 protein of three FMDV serotypes. Here BAN/JA/Ma-180/2013 (accession KJ175183); BAN/CH/Sa-304/2016 (accession MK088171) and BAN/DH/Sa-318/2018 (accession MN722609) were taken as representatives of three serotypes (O, A and Asia1, respectively). The protein structure of BAN/JA/Ma-180/2013 was colored in yellow with surface style. The structure of BAN/DH/Sa-304/2016 and BAN/DH/Sa-318/2018 were represented with blue and green-colored sticks showing protruded extrusions at sites of mutation that causes structural alteration. Conserved fragments of VP2 were represented with red color over yellow surface structure of BAN/JA/Ma-180. (B) Ramachandran plots for VP2 protein of three serotypes (O, A and Asia1) of FMDV obtained by PROCHECK. BAN/JA/Ma-180/2013 (accession KJ175183), BAN/CH/Sa-304/2016 (accession MK088171) and BAN/DH/Sa-318/2018 (accession MN722609) were used as representative. Red, yellow, beige and white represents most favored, additional allowed, generously allowed and disallowed regions, respectively. Black dots represent the amino acids of the VP2 protein.
